# Duocarmycin SA Reduces Proliferation and Increases Apoptosis in Acute Myeloid Leukemia Cells In Vitro

**DOI:** 10.3390/ijms25084342

**Published:** 2024-04-14

**Authors:** William A. Chen, Terry G. Williams, Leena So, Natalie Drew, Jie Fang, Pedro Ochoa, Nhi Nguyen, Yasmeen Jawhar, Jide Otiji, Penelope J. Duerksen-Hughes, Mark E. Reeves, Carlos A. Casiano, Hongjian Jin, Sinisa Dovat, Jun Yang, Kristopher E. Boyle, Olivia L. Francis-Boyle

**Affiliations:** 1 Department of Pharmaceutical Sciences, School of Pharmacy, Loma Linda University, Shryock Hall 24745 Stewart Street, Loma Linda, CA 92350, USA; 2 Department of Surgery, St. Jude Children’s Research Hospital, 262 Danny Thomas Place, Memphis, TN 38105, USA; 3 Department of Basic Sciences, School of Medicine, Loma Linda University, 11175 Campus Street, Loma Linda, CA 92350, USA; 4 Center for Health Disparities and Molecular Medicine, Loma Linda University, 11085 Campus Street, Loma Linda, CA 92350, USA; 5 Department of Surgery, School of Medicine, Loma Linda University, 11234 Anderson Street, Loma Linda, CA 92354, USA; 6 Center for Applied Bioinformatics, St. Jude Children’s Research Hospital, 262 Danny Thomas Place, Memphis, TN 38105, USA; 7 Departments of Pediatrics, Biochemistry and Molecular Biology, and Pharmacology, Penn State Cancer Institute, 400 University Drive, Hershey, PA 17033, USA; 8 Department of Pathology and Human Anatomy, Division of Anatomy, School of Medicine, Loma Linda University, 11175 Campus Street, Loma Linda, CA 92350, USA

**Keywords:** acute myeloid leukemia, duocarmycin SA, DNA alkylation, DNA double-strand break, apoptosis, chemotherapy

## Abstract

Acute myeloid leukemia (AML) is a hematological malignancy that is characterized by an expansion of immature myeloid precursors. Despite therapeutic advances, the prognosis of AML patients remains poor and there is a need for the evaluation of promising therapeutic candidates to treat the disease. The objective of this study was to evaluate the efficacy of duocarmycin Stable A (DSA) in AML cells in vitro. We hypothesized that DSA would induce DNA damage in the form of DNA double-strand breaks (DSBs) and exert cytotoxic effects on AML cells within the picomolar range. Human AML cell lines Molm-14 and HL-60 were used to perform 3-(4,5-dimethylthiazolyl-2)-2,5-diphenyltetrazolium bromide (MTT), DNA DSBs, cell cycle, 5-ethynyl-2-deoxyuridine (EdU), colony formation unit (CFU), Annexin V, RNA sequencing and other assays described in this study. Our results showed that DSA induced DNA DSBs, induced cell cycle arrest at the G2M phase, reduced proliferation and increased apoptosis in AML cells. Additionally, RNA sequencing results showed that DSA regulates genes that are associated with cellular processes such as DNA repair, G2M checkpoint and apoptosis. These results suggest that DSA is efficacious in AML cells and is therefore a promising potential therapeutic candidate that can be further evaluated for the treatment of AML.

## 1. Introduction

AML is a hematological malignancy that is often characterized by the arrest of hematopoietic stem cell differentiation and uncontrolled clonal expansion of immature myeloid precursors (blasts) [1]. Over time, these blasts accumulate in the bone marrow and peripheral blood, impairing the production of mature blood cells and normal hematopoiesis, and subsequently resulting in bone marrow failure [2]. While AML occurs in children and adults, it is the most common form of acute leukemia in adults with a median onset age of ~68 years at diagnosis [3]. AML is a heterogenous disease with several factors contributing to its pathogenesis. Factors include cytogenetic abnormalities, recurrent gene mutations and epigenetic/transcriptional modifications [4]. Consequently, patients present with multiple cooperating driver mutations that contribute to the AML phenotype, poor patient response to treatment and increased risk for relapse [5]. Patient risk stratification and choice of therapy take into consideration the patient’s age, identifiable comorbidities and associated adverse cytogenetics [6].

The current AML treatment regimen is divided into two phases: remission induction therapy and consolidation therapy. Standard remission induction therapy includes intravenous infusion of the cytotoxic chemotherapeutic agent cytarabine in combination with anthracyclines in a 7 + 3 regimen with the goal of achieving complete remission (CR) by reducing the accumulation of cancer cells and restoring normal hematopoiesis [7]. Induction therapy is followed by consolidation therapy, which contributes to prolonging remission in patients and reducing the risk of relapse [8]. CR is achieved using standard induction chemotherapy in 60–80% of AML patients that are less than or equal to 60 years, while CR is observed in 40–60% of patients that are older than 60 years [9]. The 3-year relapse-free and 5-year disease-free survival of patients following CR is 34.4% and 13%, respectively [10,11]. Furthermore, the rate of relapse in AML patients after achieving CR ranges from 60 to 84% [10,12]. Therefore, despite advances in the conventional standard of care, relapse among patients remains a challenge and there is a need for the investigation of therapeutic candidates that can be used in combination with current treatment regimens to reduce the risk of relapse and improve the survival rates of AML patients. 

Duocarmycins (CC-1065, duocarmycin A and DSA), a class of antitumor antibiotics that are characterized as DNA alkylating agents, have emerged as promising therapeutic candidates that are being evaluated for the treatment of cancer [13]. These agents have received attention due to their sequence-selective alkylation of DNA and their surprising cytotoxic potency [13]. Within the general class of duocarmycins, DSA is among the most promising because it (1) is considered to be among the most potent naturally occurring members of the duocarmycin class with cytotoxic IC_50_ values in the picomolar range when tested against several cancer cell lines [14,15]; (2) is solvolytically stable and remains stable outside the environs of the DNA minor groove, which increases its cytotoxic potency by alkylating only DNA within the cell; and (3) possesses DNA alkylating activity that occurs within the physiological range (pH 7.4) [16,17]. DSA was isolated from *Streptomyces* bacteria and functions as a DNA alkylator by binding to the minor groove of DNA and alkylating adenine residues at the N3 position in select adenine–thymine (AT) rich sequences [18,19]. This alkylation of adenine causes abnormal base pairing and strand breakage, resulting in DNA damage and ultimately apoptosis. The stability of the naturally occurring DSA was improved using strategies such as creating a prodrug DSA analog, namely, *seco*-DSA, with a bound chlorine to mask the cyclopropane ring within the alkylating subunit and an attached phenol functional group [20]. *seco*-DSA is converted to DSA in situ and exhibits identical DNA alkylation efficiency to the natural compound in several cancer cell lines as described by Boger et al. [21,22]. More recently, DSA has been shown to increase the sensitivity of glioblastoma multiforme cells to proton irradiation in the sub-nanomolar range in vitro [23]. In this study, we investigated for the first time the efficacy of DSA on AML cells in vitro. We hypothesized that DSA would induce DNA damage in the form of DNA DSBs and exert cytotoxic effects on AML cells within the picomolar range. Our results showed that DSA induced DNA DSBs, induced cell cycle arrest, decreased proliferation and increased apoptosis in human AML cells in a dose-dependent manner. Additionally, RNA sequencing analysis showed that DSA exerts its effects by regulating genes that are associated with DNA repair, G2M checkpoint and apoptosis. 

## 2. Results

### 2.1. Human AML Cell Lines Molm14 and HL-60 Express Myeloid Lineage Markers

**Figure 1:**
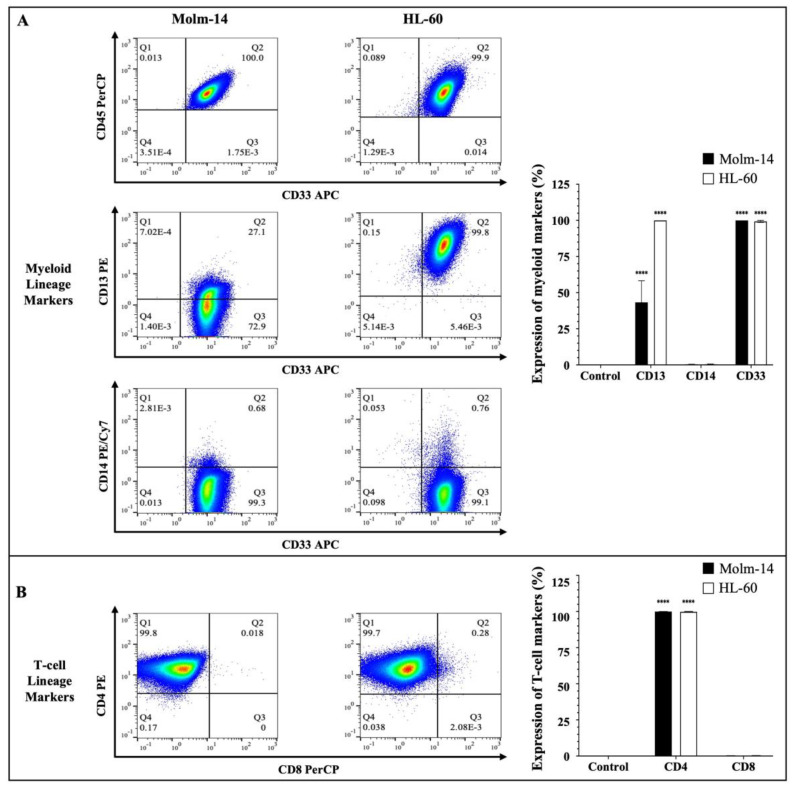
**Immunophenotyping of AML cells.** (**A**) AML cell lines (Molm-14 and HL-60) were stained with an antibody cocktail, fixed with paraformaldehyde and analyzed using flow cytometry. Shown in panel (**A**) are the representative dot plots of AML cells stained with CD45 and myeloid lineage markers CD33, CD14 and CD13, as well as bar graphs that quantify the expression of the myeloid markers. Shown in panel (**B**) are the representative dot plots and bar graphs of AML cell lines stained with T-lineage markers CD4 and CD8. Representative dot plots are one of 3 independent experiments and bar graphs are the mean ± SEM of 3 independent experiments. **** *p* < 0.0001.

Human AML cells are often characterized by the clonal expansion of immature myeloid precursors (blasts) in the bone marrow and peripheral blood [2]. In this study, we used human AML cell lines Molm-14 (established from the peripheral blood of a 20-year-old man with AML at relapse after being diagnosed initially with myelodysplastic syndrome) and HL-60 (established from a 36-year-old woman diagnosed with promyelocytic leukemia) to perform all studies [24,25]. To confirm the phenotype of these AML cell lines prior to our study, both cell lines were stained with antibodies to several hematopoietic lineage markers. Cells were stained with a pan-leukocyte marker (CD45) (Figure 1A); myeloid lineage markers (CD33, CD13 and CD14) (Figure 1A) and T-cell lineage markers (CD4 and CD8) (Figure 1B). 

The clones and sources of the antibodies that were used are summarized in Supplemental Table S1. Stem cell markers (CD34 and CD117) (Supplemental Figure S1A) and a B-cell lineage marker (CD19) (Supplemental Figure S1A) were also assessed. The cells were then analyzed using flow cytometry and the Flowjo software (version 10). The gating strategy used to analyze the data is described in Supplemental Figure S1B. Our results showed that both Molm-14 and HL-60 cells were CD45^+^, CD33^+^, CD13^+^, CD14^−^ and CD4^+^ (Figure 1A,B). Surface protein expression of the markers was quantified as shown in Figure 1A,B. Although both cell lines expressed CD13, Molm-14 cells expressed lower levels of CD13 compared to HL-60 cells (Figure 1A). Previous studies have shown that AML patients can express low levels or high levels of CD13 on the surface of their leukemic cells and patients that express low CD13 transcript levels and have low CD13 surface protein expression tend to survive longer than patients with higher CD13 levels [26]. Both cell lines did not express CD8 (Figure 1B), CD19, CD117 or CD34 (Supplemental Figure S1A). Taken together, these data confirmed that both human cell lines used in this study (1) are of the myeloid lineage based on their expression of CD45 and myeloid markers CD33 and CD13; (2) represent two categories of patients: newly diagnosed AML patients (HL-60) and relapsed AML patients (Molm-14) based on the origin of the patient samples; and (3) represent CD13 low and CD13 high AML subtypes.

### 2.2. DSA Shows Efficacy within the Picomolar Range against Human AML Cell Lines In Vitro

**Figure 2:**
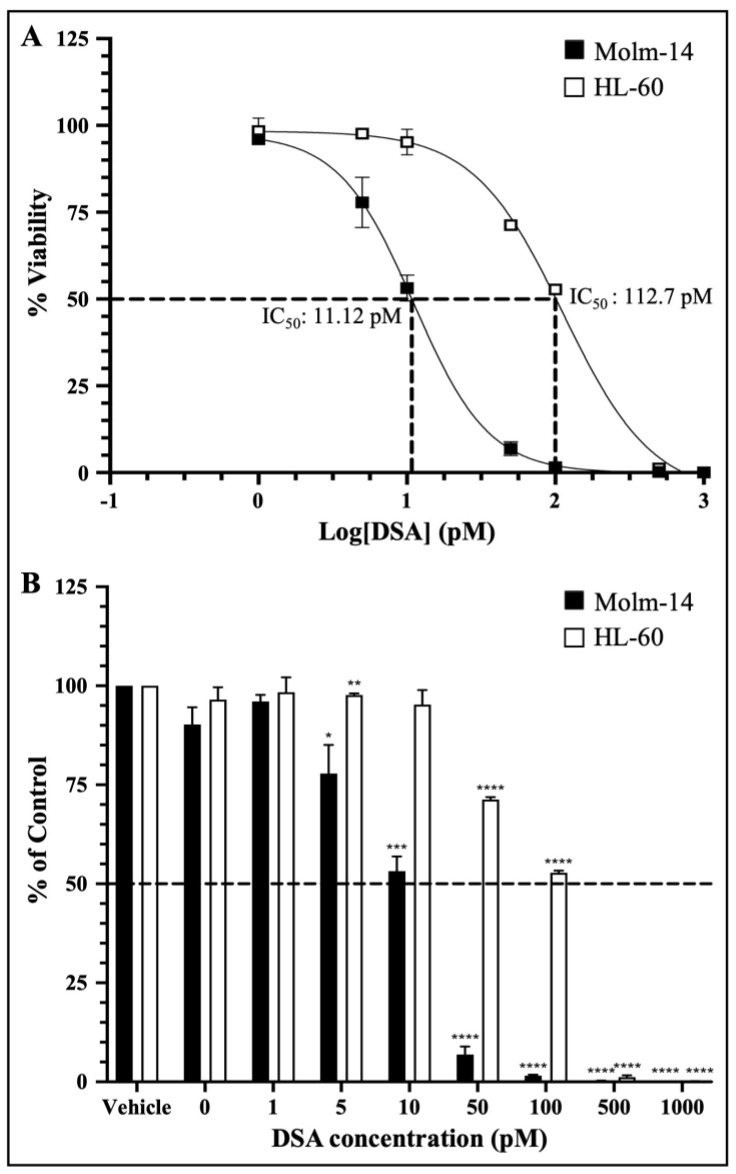
**DSA Shows Efficacy against AML Cells within the Picomolar Range.** AML cells were plated at 5000 cells per well and incubated with vehicle (DMSO) or increasing concentrations of DSA (0, 1, 5, 10, 50, 100, 500 and 1000 pM) for 72 h and analyzed using the MTT assay. Dotted lines represent half of the maximal inhibitory concentration (IC_50_) for DSA. Shown in panel (**A**) are the dose response curves for Molm-14 (black squares) and HL-60 (white squares). Graphs were plotted using GraphPad Prism and the IC_50_ was calculated using a nonlinear regression model. The results are expressed as the mean ± SEM that is representative of 3 independent experiments. Shown in panel (**B**) is a bar graph of the dose response on a linear scale with a mean ± SEM that is representative of 3 independent experiments for Molm-14 (black bars) and HL-60 (white bars). * *p* < 0.05, ** *p* < 0.01, *** *p* < 0.001, **** *p* < 0.0001.

Preclinical drug response studies using cultured cell lines are important for determining the therapeutic effectiveness of a drug. These types of studies allow for the characterization of the potency and the identification of the cellular effects of the test drug [27]. Furthermore, dose–response relationships are often used as a tool/metric to characterize the potency of a drug by providing the concentration of half the maximal inhibition (IC_50_) of the drug [27]. Therefore, to investigate whether DSA shows efficacy against AML cells, we cultured both AML cell lines with vehicle (DMSO) or seven increasing concentrations of DSA (1–1000 pM) for 72 h and analyzed the cell viability using the MTT assay (living cells undergoing respiration convert MTT to a purple formazan dye whose concentration is measured using a spectrophotometer). The data collected were analyzed using GraphPad prism, and dose–response curves for both cell lines were plotted. The IC_50_ of DSA was calculated using a nonlinear regression model. Our results showed that the IC_50_ of DSA for Molm-14 cells was 11.12 pM (Figure 2A) and the IC_50_ of DSA for HL-60 cells was 112.7 pM (Figure 2A). 

The dose–response curves for both cell lines were plotted together on a log scale showing approximately a 10-fold increase in the DSA IC_50_ for HL-60 compared to Molm-14. This observation was also summarized in the bar graph shown in Figure 2B which shows the response of both cell lines to all seven concentrations of DSA compared to vehicle using a linear scale. These data suggest that both Molm-14 and HL-60 cell lines were sensitive to DSA with IC_50_ values within the picomolar range; however, HL-60 cells seem to be more resistant to DSA compared to Molm-14 cells.

### 2.3. DSA Induces DNA Double-Strand Breaks in Human AML Cell Lines In Vitro

**Figure 3:**
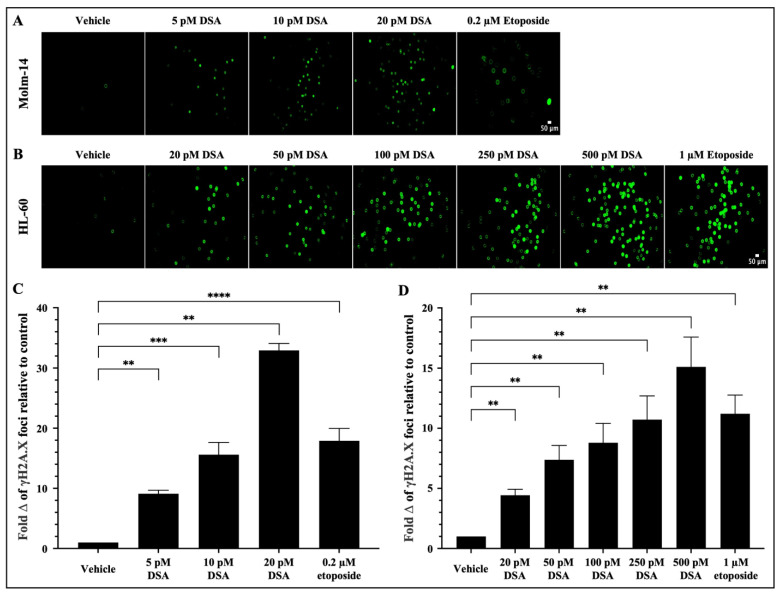
**DSA Induces DNA Double-Strand Breaks in AML cells.** AML cells (**A**, Molm-14; **B**, HL-60) were plated at 40,000 cells per well and incubated with vehicle (DMSO), etoposide or increasing concentrations of DSA. Cells were sequentially stained with an anti-phospho-histone (γH2A.X) antibody and a FITC-conjugated secondary antibody. Cells were imaged using fluorescence microscopy at a total magnification of 100×. Graphed in panel (**C**) is the fold change of γH2A.X foci in Molm-14 cells compared to vehicle (DMSO) control after treatment with DSA. Graphed in panel (**D**) is the fold change of γH2A.X foci in HL-60 cells compared to vehicle (DMSO) control after treatment with DSA. Fold change was calculated by normalizing the numerical count obtained in ImageJ for the vehicle control to one (1) and by dividing the counts obtained for each experimental group by the control to determine fold change relative to control. Data in Panels (**C**,**D**) are represented as the mean ± SEM of 3 independent experiments. ** *p* < 0.01, *** *p* < 0.001, **** *p* < 0.0001.

As a DNA alkylator, DSA’s reported mechanism of action involves its covalent binding to adenine bases in the N3 position that are found in AT rich sites within the minor groove of DNA [21]. This results in the formation of DNA adducts that are cytotoxic because they impair DNA repair pathways and result in DSBs. Following the introduction of DSBs into DNA, the variant histone H2A.X is phosphorylated at serine 139, producing phosphorylated H2AX (γH2A.X) foci at DNA damage sites [28,29]. Therefore, to evaluate whether DSA induces DSBs in our system, both AML cell lines were treated with vehicle or increasing concentrations of DSA (Molm-14 cells were treated with 5, 10 or 20 pM (Figure 3A); HL-60 cells were treated with 20, 50, 100, 250 or 500 pM (Figure 3B)) for 80 min and analyzed using the Oxiselect DNA DSB assay and fluorescence microscopy. 

Briefly, after treatment with DSA, cells were stained with the anti-γH2A.X (Ser 139) antibody, then stained with a FITC-conjugated secondary antibody and subsequently imaged using a Keyence microscope. Etoposide, a FDA-approved topoisomerase II inhibitor that is used in combination chemotherapy for relapsed/refractory AML, was used as a positive control. Our results showed that there was a significant fold increase in γH2A.X foci in Molm-14 cells (Figure 3A,C) and HL-60 cells (Figure 3B,D) after treatment with DSA in a dose-dependent manner and at picomolar concentrations compared to vehicle-treated cells. Comparatively, both cell lines treated with etoposide also showed a fold increase in the γH2A.X foci in both cell lines, but within the micromolar range. Taken together, DSA induced DSBs in both AML cell lines within the picomolar range compared to etoposide, which required micromolar concentrations to produce comparable effects. 

### 2.4. DSA Induces G2/M Cell Cycle Arrest in Human AML Cell Lines In Vitro

**Figure 4:**
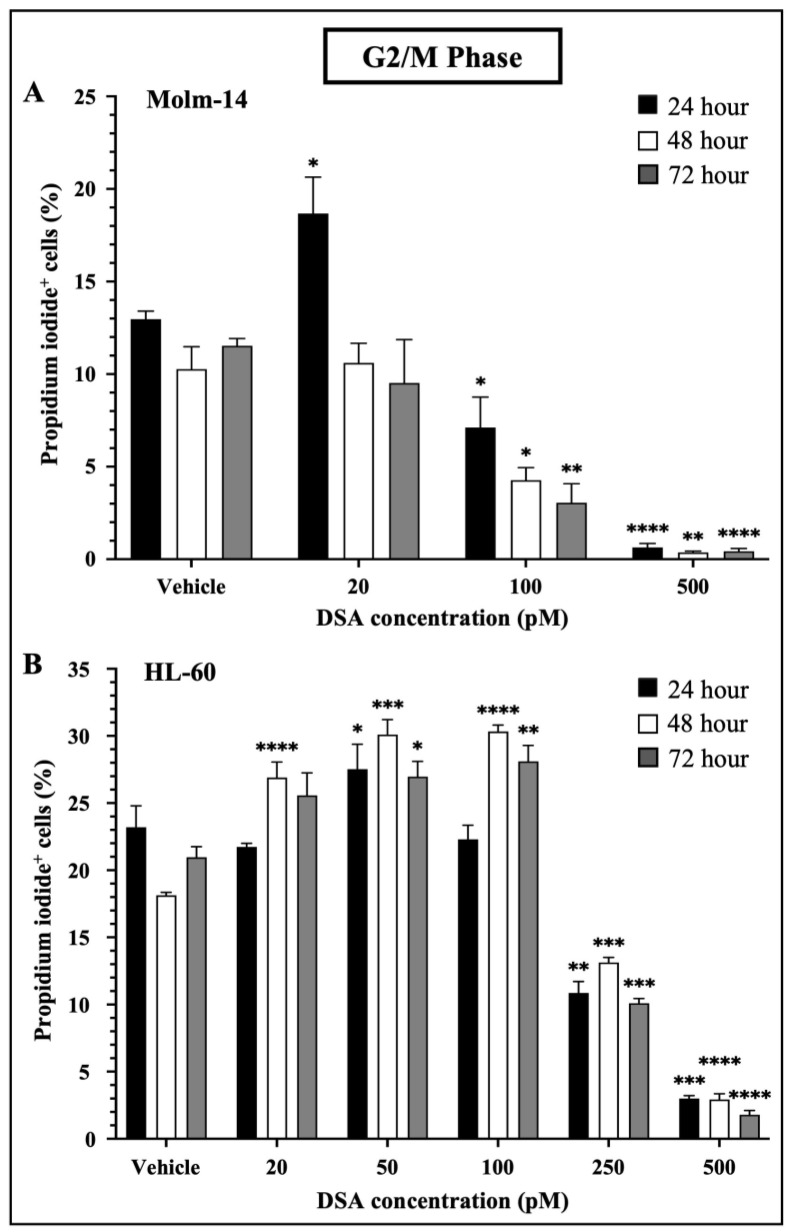
**DSA Induced Cell Cycle Arrest in AML Cells at the G2/M Phase.** Shown in panels (**A**) (Molm-14 cells) and (**B**) (HL-60 cells) are the percentages of propidium iodide^+^ cells in the G2/M phase of the cells’ cycle. Cells were incubated with vehicle (DMSO) or increasing concentrations of DSA for 24, 48 and 72 h and flow cytometry was performed. Analysis was performed using the Flowjo software and the bar graphs were generated using GraphPad Prism. Shown is the mean ± SEM that is representative of 3 independent experiments for each cell line. * *p* < 0.05, ** *p* < 0.01, *** *p* < 0.001, **** *p* < 0.0001.

When DNA DSBs occur in mammalian cells, cells activate a DNA damage checkpoint (DDC) response which leads to an arrest of the cell’s cycle, changes in gene expression and the mobilization of DNA repair mechanisms such as homologous recombination and non-homologous end joining to prevent the inheritance of unrepaired errors to daughter cells during mitosis [30]. Studies have shown that mammalian cells with as low as one to four DSBs are capable of mounting DDC responses and inducing cell cycle arrest in the G1 and G2 phases of the cell’s cycle. Moreover, when multiple DSBs occur in a G2 cell, if DNA repair is unsuccessful, most if not all of the G2 cells permanently withdraw from the cell cycle [31]. Since DSA induced multiple DSBs in the AML cell lines, we investigated whether DSA would induce cell cycle arrest in the AML cell lines. To accomplish this, both AML cell lines were treated with vehicle or increasing concentrations of DSA. Molm-14 cells were treated with 20, 100 or 500 pM DSA and HL-60 cells were treated with 20, 50, 100, 250 or 500 pM DSA for 24, 48 and 72 h. Cells were harvested, permeabilized with ethanol, stained with propidium iodide and analyzed by flow cytometry. Propidium iodide, a fluorescent dye that stains DNA, was used to quantify the DNA content of the cells in each phase of the cell cycle. The fluorescence intensity of the stained cells correlates to the amount of DNA they contain. Results from our analyses showed that DSA significantly reduced the percentage of Molm-14 cells that were in the G2/M phase at 100 pM and 500 pM of DSA at 24, 48 and 72 h (Figure 4A) while DSA significantly reduced the percentage of HL-60 cells that were in the G2/M phase at 250 pM and 500 pM of DSA at 24, 48 and 72 h (Figure 4B). 

Interestingly, there was a transient increase in the percentage of Molm-14 cells in the G2/M phase when treated with 20 pM of DSA at 24 h (Figure 4A) and there was a transient increase in the percentage of HL-60 cells when treated with 20 pM (at 48 and 72 h), 50 pM (at 24, 48 and 72 h) and 100 pM (at 48 and 72 h) of DSA (Figure 4B), suggesting that DDC responses may be occurring in an effort to repair DNA and salvage the cells. In addition to G2/M cell cycle arrest, both Molm-14 cells (Supplemental Figure S2A,B) and HL-60 cells (Supplemental Figure S2C,D) exhibited cell cycle arrest during the G0/G1 and S-phases, suggesting that DSA induces cell cycle arrest in multiple phases of the cell’s cycle and ultimately ends in a permanent arrest in the G2/M phase. An increase in the percentage of cells in sub G0 was also observed for Molm-14 cells when treated with 100 and 500 pM of DSA (Supplemental Figure S2A,B) and HL-60 cells when treated with 100, 250 and 500 pM of DSA (Supplemental Figure S2C,D), indicating that both cell lines may be shifting towards undergoing apoptosis. These findings suggest that DSA induces arrest in multiple phases of the cell’s cycle, but its effect is most pronounced within the G2/M phase. 

### 2.5. DSA Decreases the Proliferation of Human AML Cell Lines In Vitro 

**Figure 5:**
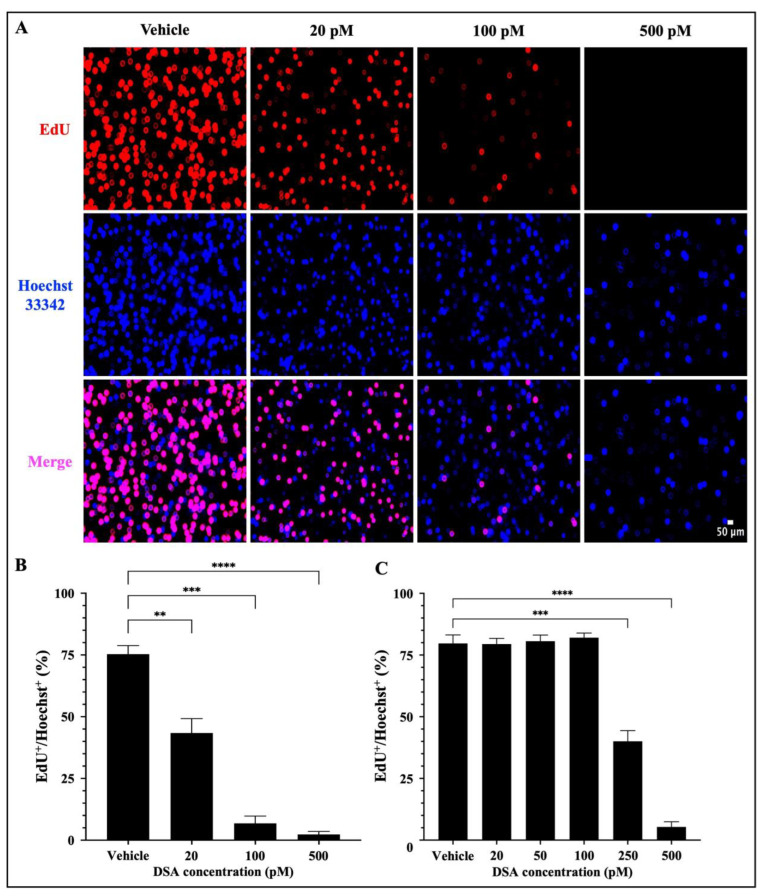
**DSA Decreases the Proliferation of AML Cells.** Molm-14 cells were plated at ~53,000 cells per well, incubated with vehicle (DMSO) or increasing concentrations of DSA for 4 days, and simultaneously treated with EdU for 3 days. Cells were harvested, fixed and permeabilized prior to sequential staining with the Alexa Fluor 647 and Hoechst 33342 dyes. Wells were imaged using fluorescence microscopy at a total magnification of 100×. Panel (**A**) shows images of Molm-14 cells that are representative of one of three independent experiments. Panel (**B**) shows a bar graph of the percentage of EdU^+^/Hoechst^+^ Molm-14 cells which was calculated by dividing the number of EdU^+^ cells by the number of Hoechst^+^ cells multiplied by 100. Data are representative of the mean ± SEM of 3 independent experiments. Panel (**C**) shows a bar graph of the percentage of EdU^+^/Hoechst^+^ HL-60 cells which was generated similarly to the data shown in panel (**B**). Data are representative of the mean ± SEM of 3 independent experiments. ** *p* < 0.01, *** *p* < 0.001, **** *p* < 0.0001.

A hallmark of AML is the uncontrolled proliferation of immature myeloid precursor cells (blasts) that accumulate in the bone marrow and contribute to (1) a reduction in normal hematopoiesis, (2) carcinogenesis and (3) metastasis [32]. Our results in Figure 4 showed that DSA-induced DSBs impact DNA synthesis in the S-phase and cause cell cycle arrest of AML cells at the G2/M phase. These data suggest that there is a reduction in DNA synthesis in the AML cells which has the potential to contribute to a reduction in cell division via mitosis, thus leading to a reduction in cellular proliferation. To further evaluate whether DSA can reduce cellular proliferation, we used the EdU click-it DNA synthesis-based cell proliferation functional assay to label and quantify newly synthesized DNA in AML cells. EdU is a nucleoside analogue of thymidine, which is incorporated into DNA during active DNA synthesis and it is fluorescently labeled with the Alexa Fluor 647 dye, which allows us to label and track the amount of EdU (synthesized DNA) in actively proliferating cells. Briefly, both AML cell lines were treated with DSA for ~96 h and simultaneously incubated with EdU for 72 h, followed by fixation and permeabilization of the harvested cells. Cells were then stained with the Alexa Fluor 647 dye, counterstained with Hoechst 33342 to confirm the presence of DNA and analyzed using fluorescence microscopy. Our results showed that DSA significantly decreased the percentage of EdU^+^ Molm-14 cells (red) alone and in the presence of Hoechst staining (pink) in a dose–dependent manner (Figure 5A,B), while DSA significantly decreased the percentage of EdU^+^ HL-60 cells at 250 pM and 500 pM of DSA (Figure 5C and Supplemental Figure S3) compared to vehicle-treated cells. 

Cells that were EdU^+^ Hoechst^+^ were producing newly synthesized DNA as evidence of proliferation. Cells that were Hoechst^+^ and EdU^−^ contained DNA, but those cells were not producing newly synthesized DNA; thus, they were not counted as proliferating cells. Altogether, DSA decreased the proliferation of both cell lines, but Molm-14 cells required lower concentrations of DSA compared to HL-60 cells to achieve similar effects. 

### 2.6. DSA Decreases the Clonogenic Capacity of Human AML Cell Lines In Vitro

**Figure 6:**
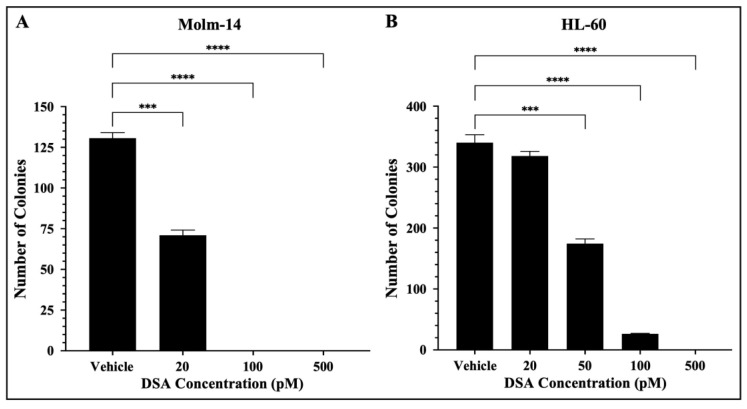
**DSA Reduces the Clonogenicity of AML cells.** AML cells (**A**, Molm-14; **B**, HL-60) were seeded at 500 cells per plate (in triplicate) in Methocult^TM^ H4435 and incubated with vehicle (DMSO) or increasing concentrations of DSA for 7 days. Colonies were counted on day 7 using an inverted light microscope. Data represent the mean ± SEM that is representative of 3 independent experiments. *** *p* < 0.001, **** *p* < 0.0001.

In addition to their ability to proliferate, leukemic cells are often characterized by their ability to generate clones leading to clonal expansion. This is of clinical significance because AML patients that undergo treatment and achieve remission can encounter relapse due to a single leukemic parent cell giving rise to a clone or a colony of cells. More importantly, this principle is the basis for the evaluation of minimal residual disease (MRD) which is often closely monitored in patients to assess the presence of clones in hopes of preventing relapse [33]. Therefore, to assess the effects of DSA on the ability of single AML cells to reproduce and survive over time to form colonies, we performed a colony forming assay using MethoCult^TM^ H4435, a methylcellulose-based medium that includes recombinant cytokines, fetal bovine serum and essential supplements that are important for the growth of human myeloid progenitor cells. Briefly, both AML cell lines were treated with vehicle or increasing concentrations of DSA, plated in MethoCult^TM^ 4435 and incubated for 7 days. Molm-14 cells were treated with 20, 100 and 500 pM of DSA, while HL-60 cells were treated with 20, 50, 100 and 500 pM of DSA. On day 7, cells were harvested and counted using an inverted light microscope. Our results showed that the number of colonies was significantly reduced by ~50% when Molm-14 cells were treated with 20 pM of DSA (Figure 6A). 

When Molm-14 cells were treated with 100 and 500 pM of DSA, there were no colonies present (Figure 6A). Additionally, the number of colonies was significantly reduced by ~50% when HL-60 cells were treated with 50 pM of DSA, and few to no colonies were formed at 100 and 500 pM of DSA (Figure 6B). Overall, the results demonstrated that DSA decreases the ability of both Molm-14 cells and HL-60 cells to form colonies over the course of several days, with HL-60 cells requiring a higher dose of DSA to reduce the colonies by 50% compared to Molm-14 cells. However, it is important to note that both the Molm-14 and HL-60 treatment groups had few to no colonies at 100 and 500 pM of DSA, suggesting that DSA might be able to overcome the resistance posed by HL-60 cells. 

### 2.7. DSA Increases Apoptosis in Human AML Cell Lines In Vitro 

**Figure 7:**
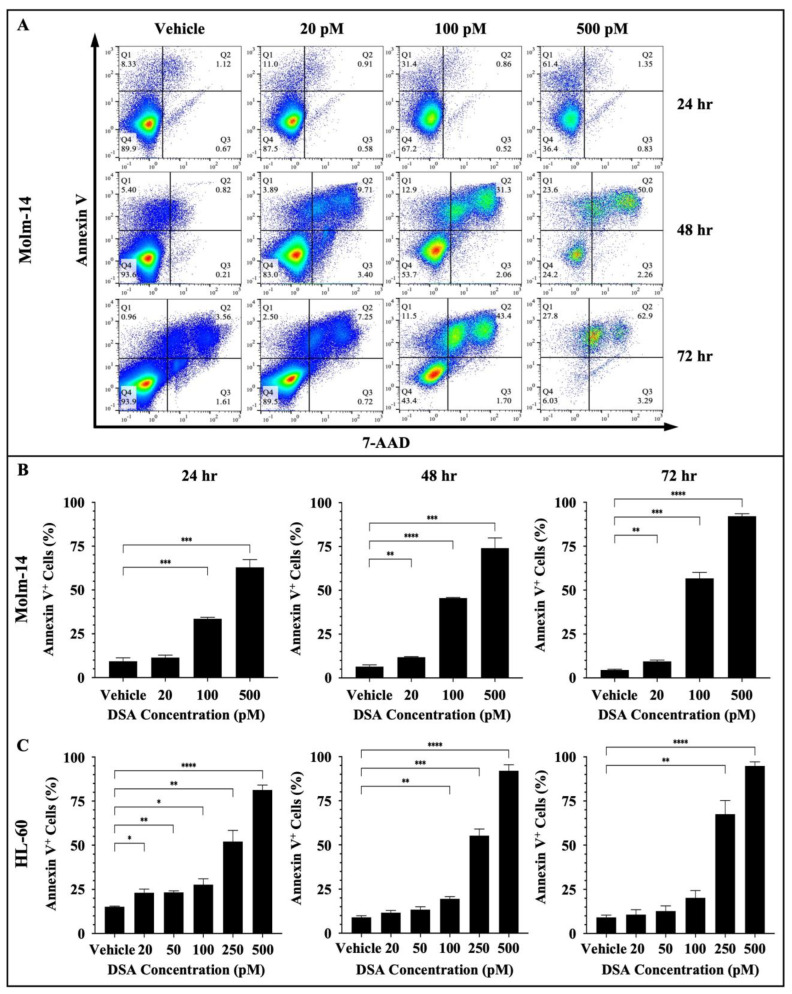
**DSA Increases Apoptosis in AML Cells.** AML cells (Molm-14 or HL-60) were incubated with vehicle (DMSO) or increasing concentrations of DSA. Cells were harvested at 24, 48 and 72 h, stained with Annexin V and 7-AAD to detect apoptotic cells and analyzed by flow cytometry. Shown in panel (**A**) are representative dot plots of early-stage (Q1: Annexin V^+^) and late-stage (Q2: Annexin V^+^7-AAD^+^) apoptotic Molm-14 cells after treatment with DSA at the various time points. Shown in panel (**B**) are bar graphs showing the percentage of apoptotic Molm-14 cells which was determined by adding the percentages of apoptotic cells in Q1 + Q2, then dividing by the total percentage (Q1 + Q2 + Q3 + Q4). Data are representative of the mean ± SEM of 3 independent experiments. Shown in panel (**C**) are bar graphs showing the percentage of apoptotic HL-60 cells which was calculated using the same method described in panel (**B**). Data are representative of the mean ± SEM of 3 independent experiments. * *p* < 0.05, ** *p* < 0.01, *** *p* < 0.001, **** *p* < 0.0001.

DNA damage often triggers DDC responses such as cell cycle arrest to repair damaged cells. However, when DNA repair mechanisms fail, multiple signals are sent throughout the intracellular compartments of the cell (such as the mitochondria) to tip the balance in favor of cell death as opposed to DNA repair and cell survival. DSBs have been shown to trigger apoptosis using multiple mechanisms. One major route is through the ATM/ATR (ataxia telangiectasia mutated/ATM and rad3-related) pathway. DSBs are detected by ATM and ATR proteins which activate downstream proteins CHK1, CHK2 and p53. p53, in turn, induces the transcriptional activation of pro-apoptotic molecules such as FAS, PUMA and BAX [30,34]. If p53 is mutated in cancer cells, then other compensatory mechanisms such as p63 and p73 mediate cell death [34]. Since DSA induced DSBs, induced cell cycle arrest and decreased the proliferative and clonogenic capacity of the AML cells, we investigated whether DSA triggers cell death via apoptosis in the AML cells. To accomplish this, both AML cell lines were treated with vehicle or increasing concentrations of DSA for 24, 48 and 72 h. Molm-14 cells were treated with 20, 100 or 500 pM of DSA, while HL-60 cells were treated with 20, 50, 100, 250 or 500 pM of DSA. Cells were harvested at the various time points, stained with the apoptotic markers Annexin V and 7-AAD and analyzed by flow cytometry and the Flowjo software (version 10). Our results showed a significant increase in the percentage of Annexin V^+^ Molm-14 cells when they were treated with 100 and 500 pM DSA at 24 h and when they were treated with 20, 100 and 500 pM DSA at 48 and 72 h (Figure 7A,B). 

We also observed a significant increase in the percentage of Annexin V^+^ HL-60 cells when they were treated with 20, 50, 100, 250 or 500 pM DSA at 24 h; when they were treated with 100, 250 or 500 pM DSA at 48 h; and when they were treated with 250 and 500 pM DSA at 72 h (Figure 7C and Supplemental Figure S4A). In summary, both cell lines showed a significant increase in the percent of apoptotic cells; however, Molm-14 cells had undergone both early- and late-stage apoptosis (Figure 7A), while HL-60 cells had undergone only early-stage apoptosis (Supplemental Figure S4A). Cells that were undergoing early-stage apoptosis were Annexin V^+^ only (Q1 in dot-plots); cells that were undergoing late-stage apoptosis were Annexin V^+^7-AAD^+^ (Q2 in dot-plots). This data suggests that DSA may be inducing apoptosis as the primary mechanism of cell death in Molm-14 cells, while DSA may be inducing different cell death mechanisms in addition to apoptosis in HL-60 cells.

Next, both AML cell lines were treated with vehicle or increasing concentrations of DSA and imaged using Hoffman Modulation Phase Contrast Microscopy at 6, 24, 48 and 72 h to identify morphological changes that occur as evidence of apoptosis. Briefly, Molm-14 cells were treated with 20, 100 or 500 pM DSA, while HL-60 cells were treated with 20, 50, 100, 250 or 500 pM DSA. At each time point, the cells were removed from the incubator and visualized on the microscope, and images were taken to identify morphological changes. Our results showed that at 6 h of DSA treatment, no differences in cell morphology were observed in Molm-14 cells; however, at 24, 48 and 72 h, we observed an increase in the morphological changes of the Molm-14 cells at higher concentrations of DSA (Supplemental Figure S4B). The morphological changes included apoptotic blebs (green arrows), ghost membranes (red arrows) and cell shrinkage (blue arrows) (Supplemental Figure S4B). Similarly, an increase in the morphological changes of the HL-60 cells was observed only at higher concentrations of DSA (Supplemental Figure S4C). Taken together, the data suggest that DSA increases apoptosis in both AML cell lines with Molm-14 cells undergoing both early- and late-stage apoptosis at lower concentrations of DSA and HL-60 cells undergoing only early-stage apoptosis at higher concentrations of DSA compared to Molm-14 cells.

### 2.8. DSA Regulates Genes That Are Associated with G2M Checkpoint, DNA Repair and Apoptosis in AML Cell Lines In Vitro

**Figure 8:**
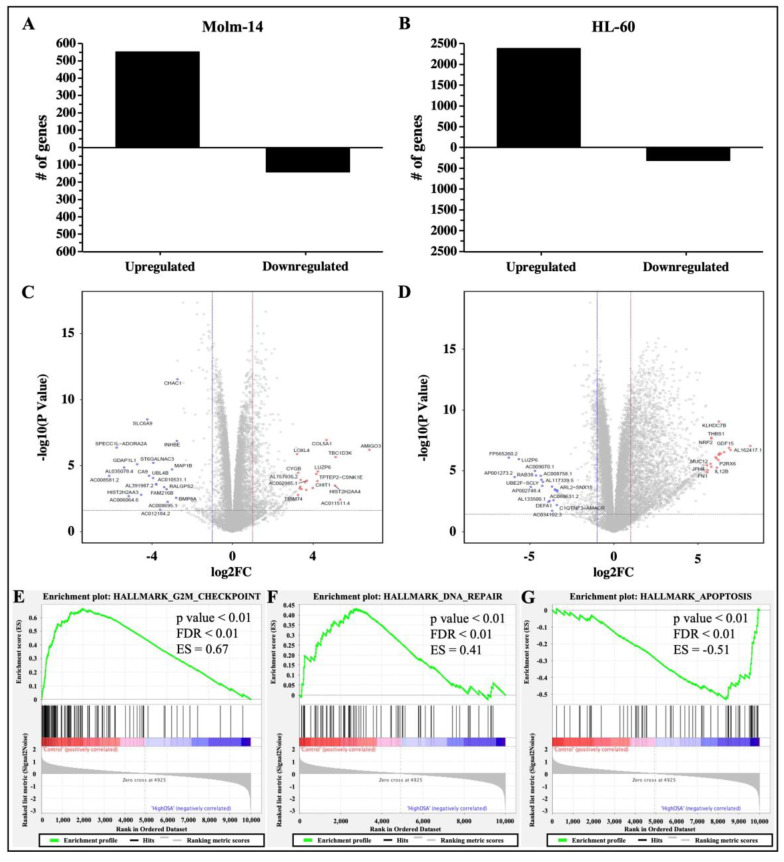
**RNA Sequencing Analysis of AML Cells Treated with DSA.** AML cells were plated at 6.0 × 10^5^ cells per well and incubated with DSA (Molm-14: 100 pM DSA; HL60: 500 pM DSA) or without DSA. Cells were harvested at 36 h and RNA was isolated. Differential gene expression was modeled using the voom method. The bar graphs represent the number of genes that were upregulated or downregulated (fold change > 2, *p*-value < 0.05) in Molm-14 (**A**) and HL-60 (**B**) cells treated with DSA compared to untreated controls. The volcano plots represent the top 20 statistically significant genes that were upregulated (red dots) or downregulated (blue dots) in Molm-14 (**C**) and the top 20 statistically significant genes that were upregulated (red dots) or downregulated (blue dots) in HL-60 (**D**) cells. The horizontal dashed lines represent the statistical significance threshold (adjusted *p*-values ≤ 0.05) and the two vertical dashed lines represent the threshold of log2 fold-change ≥1 and ≤−1 for both volcano plots. (**E**–**G**) GSEA revealed three overlapping pathways (G2M Checkpoint, DNA-repair and Apoptosis) that were significantly enriched upon treatment with high DSA (HL-60 and Molm-14 treated with 100 pM and 500 pM respectively) compared to controls (HL-60 and Molm-14 untreated). The *p*-value, FDR value and enrichment scores (ES) are included in each enrichment plot.

Studies have shown that DNA DSBs have the ability to induce gene expression changes that lead to cell cycle arrest, activate DNA repair mechanisms to fix the DNA damage in cells, or activate cell death mechanisms as an alternative if DNA repair mechanisms are unsuccessful in damaged cells [30]. Therefore, to determine the gene expression profile and identify differentially expressed genes and associated pathways that are activated by DSA to achieve the DSA-induced functional effects that we have observed in the AML cells, both cell lines were treated with DSA and RNA sequencing and analyses were subsequently performed. Briefly, Molm-14 cells were cultured in growth media with or without 100 pM DSA and HL-60 cells were cultured in growth media with or without 500 pM DSA for 36 h. At 36 h, the cells were harvested, and RNA was isolated and sent for RNA sequencing and analyses. Results from the differential gene expression analyses showed that there were 552 genes that were upregulated and 141 genes that were downregulated (with a fold change > 2 and a *p*-value < 0.05) in DSA-treated Molm-14 cells compared to controls (Figure 8A), while there were 2386 genes that were upregulated and 316 genes that were downregulated (with a fold change > 2 and a *p*-value < 0.05) in DSA-treated HL-60 cells compared to controls (Figure 8B). 

The top 20 genes that were upregulated in both cell lines and the top 20 genes that were downregulated in both cell lines are represented in volcano (scatter) plots showing the statistical significance (−log10 *p*-value) and magnitude of change (log 2-fold change) for each gene (Figure 8C,D). A list of the ID, symbol and log2 fold change for each gene is provided in Supplemental Table S2A–D. The functions of several of the top genes that were differentially expressed are not well understood; however, the functions of some dysregulated genes have been previously described as they relate to cancer and may correlate with DSA-induced cellular processes described here. For example, in Molm-14 cells, the top 20 upregulated genes included *GALR2* (encodes Galanin receptor 2, a G-protein-coupled receptor that decreased proliferation and induced apoptosis in human head and neck cancer cells and human neuroblastoma cells [35,36]) and *CYGB* (a member of the globin superfamily, which was shown to reduce proliferation in human breast cancer cells, human colorectal cancer cells, human pancreatic cancer and human lung adenocarcinoma, as well as apoptosis in human breast cancer cells [37,38,39,40]). The top 20 downregulated genes included *BMP8A* (a member of the transforming growth factor-β (TGF-β) family that promoted proliferation and inhibited apoptosis of renal cell carcinoma [41]) and *MAP1B* (a member of the MAP family that plays a role in the proliferation and invasive abilities of urothelial carcinoma cells [42]). In HL-60 cells, the top 20 upregulated genes included *NUPR1* (a transcription factor that was involved in regulating apoptosis and DNA repair in cervical cancer cells [43,44]) and *THBS1* (an extracellular glycoprotein that induced apoptosis in granulosa cells and induced apoptosis in the endothelial cells of mice with melanoma [45,46,47]). The top 20 downregulated genes included *RAB38* (a GTPase of the Ras superfamily that promoted cell proliferation in human bladder cancer cells [48]) and *LUZP6* (a cryptic tumor antigen that triggered antitumor immune responses in patients with myeloproliferative diseases [49]). 

To further delineate the mechanisms by which DSA performs its functions in AML cells, we performed gene set enrichment analysis (GSEA) to identify sets of genes that have been enriched and pathways that have been activated in the DSA treatment groups compared to controls for both cell lines. Our results showed that the hallmark gene sets that were enriched included G2M checkpoint, DNA-repair and apoptosis. Enrichment plots comparing Molm-14 and HL-60 samples that were treated with high concentrations of DSA to their respective controls showed that there were fewer genes (black lines) involved in G2M checkpoint (Figure 8E) and DNA repair (Figure 8F) processes in DSA treatment groups compared to controls. In contrast, more genes were involved in apoptosis in the DSA treatment groups compared to controls (Figure 8G). To identify individual genes that were associated with the three hallmark gene sets and their expression patterns, heat maps listing the genes in each gene set were constructed and shown (Supplemental Figure S5A–C). Several genes associated with G2M checkpoint and DNA repair functions were downregulated (see highlighted genes in blue boxes in Supplemental Figure S5A,B), while several genes associated with apoptosis functions were upregulated (see highlighted genes in red boxes in Supplemental Figure S5C). Taken together, these data suggest that DSA’s mechanisms in these cells may be mediated by downregulating genes that control G2M checkpoint and DNA repair mechanisms and upregulating genes that increase apoptosis.

### 2.9. DSA Regulates Genes That Modulate Drug Sensitivity and Chemoresistance in AML Cell Lines In Vitro

**Figure 9:**
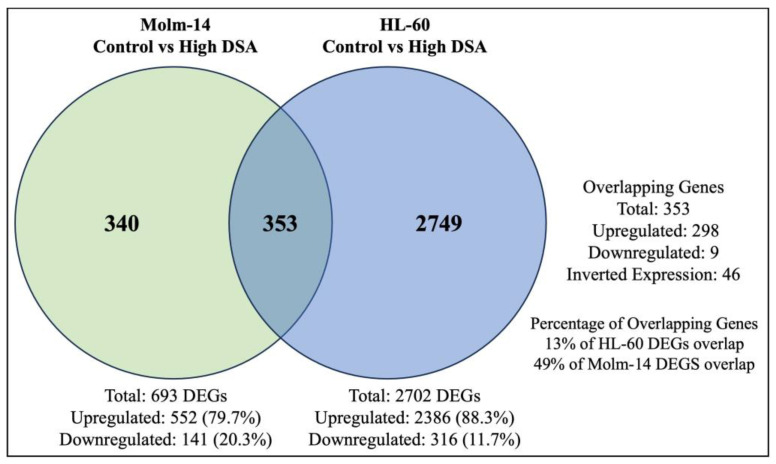
**DSA-induced Differentially Expressed Genes that Overlap between Molm-14 and HL-60 Cells.** The Venn diagram shows the number of genes that are unique to Molm-14 cells (340), unique to HL-60 cells (2749) and overlapping between both cells (353). The significance threshold was set at fold change >2 and *p*-value < 0.05.

Many anti-cancer agents are designed to cause the death of cancer cells by inducing DNA damage [50]. However, in response, many cancer cells may initiate DNA repair pathways to resist the anti-cancer agents and keep the cancer cells alive, leading to tumor progression [50]. Results from our study showed that Molm-14 cells were more sensitive to DSA (lower IC_50_), while HL-60 cells were more resistant to DSA (higher IC_50_). Therefore, to determine why HL-60 cells were more resistant to DSA compared to Molm-14 cells, first, we further analyzed the RNA sequencing data to identify the differentially expressed genes that are unique to Molm-14 cells, unique to HL-60 cells, and similar (overlapping) in both cell lines after treatment with DSA. To summarize these data, we created a Venn diagram that displays the number of differentially expressed genes that are unique and overlapping between the cell lines (Figure 9).

Further analyses of the overlapping genes showed that several of the top 20 genes that were upregulated in HL-60 cells were associated with chemoresistance (Table 1). Some of these genes were also upregulated in Molm-14 cells; however, they were more significantly upregulated in HL-60 cells than in Molm-14 cells. Select genes that were found to be associated with chemoresistance in other cancers included *GDF15* [51], *THBS1* [52], *CDKN1A* [53] and *CLU* [54] (Table 1). The log2 fold change value for each gene is provided in the table. In summary, our results showed that several genes that are associated with chemoresistance are more significantly upregulated in HL-60 cells compared to Molm-14 cells, which can help to explain the increased drug resistance that was observed in HL-60 cells after treatment with DSA. 

Second, we conducted further analyses on the GSEA data to determine whether there were DNA repair genes that were differentially expressed between the untreated groups (untreated HL-60 cells compared to untreated Molm-14 cells) prior to DSA treatment and between the treatment groups (treated HL-60 cells compared to treated Molm-14 cells) after treatment with DSA. To accomplish this, we reviewed the list of genes in the GSEA heat map (Supplemental Figure S5B) and identified DNA repair genes that were differentially expressed (red vs. blue) between the HL-60 and Molm-14 untreated groups (Supplemental Table S3—left) and DNA repair genes that were differentially expressed between the HL-60 and Molm-14 treatment groups (red vs. blue) (Supplemental Table S3—right). Our results showed that in the untreated groups, several DNA repair genes were expressed by both cell lines, with similar expression levels, while several DNA repair genes were differentially expressed. Four (4) select DNA repair genes that were differentially expressed by both cell lines prior to treatment were *POLR2E, POLR2C, POLR2K* and *POLB* (Supplemental Table S3—left), suggesting that although both cell lines may share some common DNA repair genes, each cell line also utilizes unique or different DNA repair genes (and by extension different DNA repair pathways) even prior to their treatment with DSA. 

Analyses of the data after DSA treatment showed that *POLR2C, POLR2K* and *POLB* expression patterns in both cell lines did not change after treatment with DSA; however, several DNA repair genes were differentially expressed in HL-60 compared to Molm-14 after treatment with DSA (Supplemental Table S3—right). A closer look at these genes showed that there were nine (9) select DNA repair genes that were not significantly differentially expressed in the untreated cells prior to DSA treatment, but which were differentially expressed between the two cell lines after treatment with DSA (Supplemental Table S3—right). Of these nine (9) genes, six (6) (*RFC5, RFC2, PCNA, RPA2, RAD51* and *FEN1*) were upregulated in HL-60 cells (red), suggesting that after treatment with DSA, HL-60 cells may be upregulating DNA repair genes in order to repair the DNA damage caused by DSA, thus explaining the chemoresistance observed in these cells compared to Molm-14 cells. Conversely, these six (6) genes were downregulated (blue) in Molm-14 cells, suggesting that these DNA repair processes may be absent or significantly reduced, rendering Molm-14 cells more sensitive to DSA. Further analyses of these genes using GeneCards (“Pathways”) showed the DNA repair pathways that are associated with each gene. The pathways included base excision repair (BER), nucleotide excision repair (NER), mismatch repair (MMR), homologous recombination (HR) and non-homologous end joining (NHEJ) (Supplemental Table S3). In summary, the expression patterns of these six (6) genes provide insights about which DNA repair genes and associated pathways may be involved in influencing AML cell resistance and AML cell sensitivity to DSA. Therefore, the expression patterns of these genes may be used to predict AML cellular responses to DSA. 

## 3. Discussion

**Figure 10:**
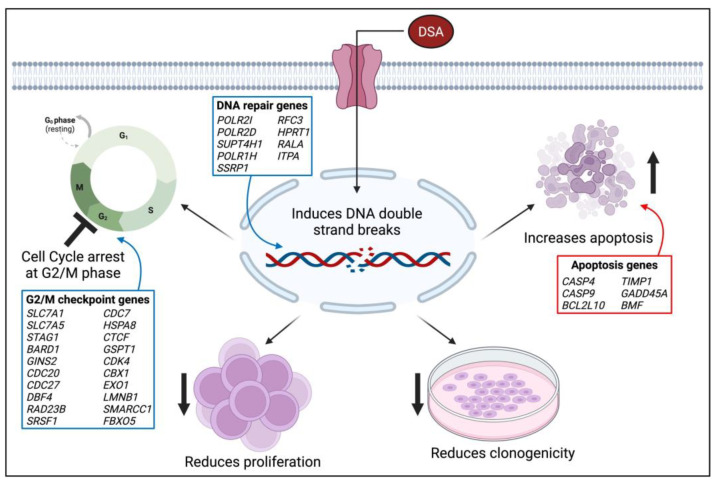
**Model of DSA’s Mechanism of Action on AML Cells.** The proposed model summarizes the functional effects of DSA on AML cells in vitro. The blue boxes represent genes that are downregulated and the red boxes represent genes that are upregulated. The image was created using BioRender.com. Symbols: ↑—increase; ↓—reduce; 丄—inhibit.

Since its initial isolation, synthesis and evaluation by the Ichimura and Boger groups, DSA and its congeners have been investigated in several types of solid tumors including small cell lung cancer, uterine cancer, ovarian cancer and glioblastoma multiforme [23,55,56]. However, DSA has not been investigated in blood cancers, or AML specifically. In this study, we evaluated the efficacy of DSA in AML cells in vitro. Results from our study showed that DSA induced DNA DSBs and induced cell cycle arrest at the G2M phase in AML cells. DSA also reduced the proliferative and clonogenic capacities of the AML cells and increased apoptosis in these cells. The effects of DSA on the above-mentioned cellular processes are supported by DSA’s regulation of genes that are known to be involved in G2M checkpoint, DNA repair and cellular apoptosis. DSA downregulated genes that are known to control/enhance G2M checkpoint and DNA repair, while upregulating genes that are known to induce cellular apoptosis. A proposed model that summarizes the effects of DSA in the AML cells is provided in Figure 10. These results provide for the first time a description of the efficacy of DSA in AML cells, and a description of the cellular and genetic mechanisms by which DSA accomplishes its effects.

It is important to note that these DSA-mediated effects in AML cells were achieved using picomolar concentrations of DSA. A potential explanation for its potency is its ability to produce DNA damage that is highly focused in the minor groove of DNA at the N3 position of adenine, and the difficulty encountered by cellular DNA repair mechanisms to repair the damage produced by DSA [21]. DSA’s potency is of significance because most chemotherapeutic agents that are currently used for the treatment of AML have IC_50_s in the nanomolar and micromolar range [57]. Earlier studies evaluated the efficacy of a duocarmycin derivative (KW-2189) compared to other clinically active drugs in vivo against several solid tumors [58]. These drugs included cisplatin, mitomycin C, Adriamycin and cyclophosphamide [58]. Cyclophosphamide has been FDA-approved for the treatment of AML. Results from the study showed that KW-2189 was more efficacious than these other drugs in vivo in murine and xenograft models, thus highlighting DSA as a more potent and effective drug to be considered for further evaluation as a potential therapeutic candidate for the treatment of AML alone or in combination with other FDA approved drugs. Besides the benefit of lower IC_50_ values and its potency, DSA has the potential to (1) be combined with other chemotherapeutic agents at lower therapeutic doses; (2) be combined with targeted therapies to increase selectivity and reduce toxicity; and (3) reduce the time to transplant by potentially inducing early remission and reducing relapse.

Because of its cytotoxic potency within the picomolar range, DSA is also being considered in the biotherapeutic field as a viable candidate to be used as a payload for the construction of antibody drug conjugates (ADCs) for use in other types of cancers [59]. For example, DSA has been used to construct an ADC, Promiximab-DUBA (an anti-CD56 monoclonal antibody conjugated to duocarmycin), for the treatment of small cell lung cancer (SCLC) [56]. Promiximab-DUBA exerted inhibitory effects on SCLC cells in vitro and in vivo in SCLC xenograft models, thus introducing a new approach for the treatment of SCLC [56]. Currently, there are no FDA-approved DSA-ADCs; however, a number of DSA-ADCs are currently in clinical trials for different cancers other than AML [60]. Further investigation of the potential use of DSA as an ADC for the treatment of AML is warranted. 

Although DSA showed efficacy within the picomolar range in both AML cell lines, it is noted that Molm-14 cells were more sensitive to DSA than HL-60 cells. A possible explanation for this is that HL-60 cells may be utilizing compensatory mechanisms such as upregulating DNA repair genes and associated pathways and downregulating apoptotic mechanisms when treated with lower concentrations of DSA. In support of this, our results showed that HL-60 cells may be upregulating chemoresistance mechanisms via DNA repair pathways to increase cancer cell survival. However, as DSA’s concentration increases, over time the scales seem to tip so that HL-60 cells undergo apoptosis. Additionally, results from a previous study suggest that the difference in CD13 surface protein expression impacts the survival outcomes in AML patients [26] and thus may explain the difference in response to treatment that was observed in both cell lines. 

A limitation of this study is that primary samples were not evaluated in this study. Therefore, future studies will focus on (1) evaluating the efficacy of DSA using primary samples in in vivo models; (2) investigating the molecular mechanisms by which DSA induces cell death in AML cells; (3) elucidating the resistance mechanisms that are employed by some AML cells in response to treatment with DSA; and (4) evaluating the efficacy of DSA in combination with other therapeutic candidates. 

## 4. Materials and Methods

### 4.1. Human Cell Lines 

Human AML cell lines Molm-14 and HL-60 were obtained from the Payne Laboratory (Loma Linda University, Loma Linda, CA, USA). Molm-14 cells were routinely cultured in R10 media containing RPMI 1640 GlutaMAX™ (Gibco, Thermo Fisher Scientific, Waltham, MA, USA) supplemented with 10% heat-inactivated (HI) fetal bovine serum (FBS, Gibco, Thermo Fisher Scientific, Waltham, MA, USA), L-glutamine, 100 IU/mL penicillin and 0.1 mg/mL streptomycin. HL-60 cells were routinely cultured in media containing IMDM (Gibco, Thermo Fisher Scientific, Waltham, MA, USA) supplemented with 20% HI FBS (Gibco, Thermo Fisher Scientific, Waltham, MA, USA), L-glutamine, 100 IU/mL penicillin and 0.1 mg/mL streptomycin. All cells were maintained in T75 flasks (Celltreat Scientific, Shirley, MA, USA) and incubated in 5% CO_2_ at 37 °C. Cells were passaged every 2–3 days once ~80% confluency was observed to maintain healthy, viable cells. 

### 4.2. Flow Cytometry

Cells were harvested from culture and stained with the fixable viability dye (FVD) eFluor 450 (eBioscience, Thermo Fisher Scientific, Waltham, MA, USA) to label viable cells and a panel of hematopoietic lineage markers to immunophenotype the myeloid leukemia cells. Cells were then incubated with the specific anti-human antibody clones to the lineage markers on ice for 15 min. Samples were analyzed on the same day using the MACSQuant Analyzer 10 and Flowjo software version 10. Analyses were conducted using doublet discrimination and controls including unstained, isotype control and fluorescence minus one (FMO) samples. 

### 4.3. MTT Dose Response Assay

DSA-induced growth inhibition in the AML cell lines was evaluated using the 3-(4,5-dimethylthiazol-2-yl)-2,5-diphenyltetrazolium bromide (MTT) Cell Proliferation Kit I (Roche Diagnostics, Mannheim, Germany) according to the manufacturer’s instructions. Molm-14 cells were harvested from culture and seeded in a 96-well cell culture plate at a density of 5 × 10^3^ cells/well and treated with vehicle (DMSO) or increasing concentrations of DSA (0–1000 pM) for 72 h in a 5% CO_2_ incubator at 37 °C. HL-60 cells were harvested from culture and seeded in a 96-well cell culture plate at a density of 5 × 10^3^ cells/well and treated with vehicle (DMSO) or increasing concentrations of DSA (0–1000 pM) for 72 h. Subsequently, the cells were labeled with the MTT labeling reagent for 4 h followed by the addition of a solubilization buffer for ~12–16 h (overnight). The plate was then read using the μQuant microplate spectrophotometer (BioTek^®^ Instruments, Inc., Winooski, VT, USA) and the absorbance was read at 570 nm wavelength using 650 nm as the reference wavelength.

### 4.4. DNA Double-Strand Break Assay

DNA double-stranded breaks (DSBs) were assessed using the OxiSelect DNA Double-Strand Break (DSB) Staining Kit (Cell Biolabs, San Diego, CA, USA) according to the manufacturer’s instructions with slight modifications. Briefly, 4 × 10^4^ cells/well were seeded in a flat-bottom 96-well plate and incubated overnight. Cells were then treated with DMSO, etoposide or different concentrations of DSA for 80 min. Cells were subsequently fixed with 3.7% formaldehyde for 15 min at room temperature in the dark, washed with PBS, permeabilized with ice cold 90% methanol for 10 min at 4 °C in the dark and washed with PBS. Finally, cells were incubated with blocking buffer containing 1% bovine serum albumin (Sigma-Aldrich, MI, USA) diluted in PBS for 30 min at room temperature on an orbital shaker, then stained with an anti-γ-H2A.X antibody for 1 h at room temperature on an orbital shaker and stained with a fluorescein isothiocyanate (FITC)-conjugated secondary antibody for 1 h at room temperature on an orbital shaker. Fluorescence imaging of γH2A.X was conducted using the Keyence BZ-X710 microscope (Keyence Corporation, Osaka, Japan) at a total magnification of 400×.

### 4.5. Cell Cycle Assay

Molm-14 cells and HL-60 cells were seeded at a density of 3.0 × 10^5^ cells per well and 1.5 × 10^5^ cells per well, respectively, in a 6-well plate and treated with vehicle or increasing concentrations of DSA for 24, 48 and 72 h. Cells were harvested, permeabilized with ice cold 70% ethanol (Fisher Scientific, Fair Lawn, NJ, USA), incubated with 10 µg/mL ribonuclease (RNase-Qiagen, Hilden, Germany) for 15 min at room temperature and then stained with PI (Biolegend, San Diego, CA, USA) for 30 min in the dark at 37 °C. All samples were analyzed on the MACSQuant 10 the same day and the data were analyzed with Flowjo version 10.

### 4.6. Cell Proliferation Assay

Cellular proliferation was assessed using the Click-iT EdU Cell Proliferation Kit for Imaging (Alexa Fluor 647 dye) according to the manufacturer’s instructions (Thermo Fisher Scientific, Waltham, MA, USA) with slight modifications. Briefly, Molm-14 and HL-60 cells were seeded at a density of ~53,000 cells/well in a 4-well Lab-Tek II Chamber overnight (Nunc, Thermo Fisher Scientific, Waltham, MA, USA). Cells were treated with DSA, then labeled with 5 μM 5-ethynyl-2′-deoxyuridine (EdU) for 72 h in a 5% CO_2_ incubator at 37 °C. Subsequently, cells were harvested, fixed with 3.7% formaldehyde for 15 min at room temperature in the dark, permeabilized with ice cold 70% ethanol for 180 min (30 min at 4 °C and 150 min at −20 °C) in the dark, stained with the Click-iT reaction cocktail containing the Alexa Fluor 647 dye for 30 min at room temperature in the dark to fluorescently label EdU^+^ cells and washed with PBS. DNA was then counterstained with 5 ug/mL Hoechst 33342 for 30 min at room temperature in the dark. EdU and Hoechst 33342 labeled cells were imaged using fluorescence microscopy on the Keyence BZ-X710 microscope (Keyence Corporation, Osaka, Japan) at a total magnification of 100×.

### 4.7. Apoptosis Assay

Cellular apoptosis was evaluated using the FITC Annexin V Apoptosis Detection Kit with 7-AAD (Biolegend, San Diego, CA, USA). Cell counts were used to determine the volume of cell suspension needed to seed 3.0 × 10^5^ Molm-14 cells per well or 1.5 × 10^5^ HL-60 cells per well in a 6-well plate. Cells were then treated with vehicle or increasing concentrations of DSA for 24, 48 or 72 h. Cells were harvested, washed, resuspended in Annexin V binding buffer and stained with Annexin V for 15 min at room temperature in the dark. Subsequently, cells were washed, resuspended in Annexin V binding buffer, stained with 7-AAD (Biolegend, San Diego, CA, USA) for 15 min at room temperature in the dark, washed and resuspended in 1% paraformaldehyde (PFA) for flow cytometry analysis. All samples were analyzed on the MACSQuant 10 the same day and the data were analyzed with Flowjo version 10.

### 4.8. Colony Formation Assay

Molm-14 and HL-60 cells were seeded at 5 × 10^2^ cells, treated with vehicle or increasing concentrations of DSA (0.02, 0.1 and 0.5 nM) and were plated in methylcellulose (Methocult H4435, Stem Cell Technologies, Vancouver, BC, USA) in 35 mm cell culture dishes (Trueline, Baton Rouge, LA, USA). The dishes were incubated in a 5% CO_2_ incubator at 37 °C for 7 days and the number of colonies were counted using an inverted light microscope (Labforce, Swedesboro, NJ, USA).

### 4.9. Fluorescence Microscopy

Fluorescent images were captured on the Keyence BZ-X710 microscope (Keyence Corporation, Osaka, Japan) with a 10× objective lens and the BZ-X analyzer software version 1.4.0.1. For the cell proliferation assay involving EdU Alexa Fluor 647 and Hoechst 33343 staining, the Cy5 and DAPI filter cubes were used, respectively. For the DSB assay involving γH2A.X FITC staining, the GFP filter cube was used. Images were analyzed using the Fiji ImageJ software version 1.53t (National Institutes of Health, Bethesda, MD, USA) and the data were quantified using GraphPad Prism version 10 (GraphPad Software Inc., San Diego, CA, USA) and displayed as graphs. 

### 4.10. Statistical Analyses

Statistical analyses were performed using GraphPad Prism 10 (GraphPad Software Inc., San Diego, CA, USA). Experimental group comparisons were evaluated using the two-tailed, unpaired *t*-test in GraphPad InStat Prism version 10 (GraphPad Software Inc., San Diego, CA, USA). Statistical significance was determined by a *p*-value that is ≤0.05. All data are representative of three independent experiments and presented as the mean ± standard error of mean (SEM). * *p* < 0.05, ** *p* < 0.01, *** *p* < 0.001, **** *p* < 0.0001.

### 4.11. Bulk RNA-Seq and Data Analysis: Cell Treatment, Sequence Alignment, Differential Gene Expression and Gene Set Enrichment Analyses 

Molm-14 and HL-60 cells were seeded at 6.0 × 10^5^ cells in a 6-well plate and treated with vehicle or DSA for 36 h. Cells were harvested and RNA was isolated from the cells using Qiagen’s RNeasy mini kit. RNA concentration and quality was determined using the Nanodrop 2000c spectrophotometer (Thermo Scientific, Waltham, MA, USA). RNA sequencing and gene expression profiling analysis were performed by the Hartwell Center for Biotechnology and the Center for Applied Bioinformatics at St. Jude Children’s Research Hospital, respectively. Additional analyses were performed by collaborators in the Department of Surgery at STJCRH and the Center for Health Disparities and Molecular Medicine at Loma Linda University.

Briefly, mouse and human sequences were mapped to the hg38 genomes using the STAR aligner (v 2.7) [61]. Gene level quantification was determined using RSEM (v1.31) [62] and based on GENCODE annotation (Human Release 31). Non-coding and GENCODE level 3 genes were excluded. Differential gene expression was modeled using the voom method [63], available in the Limma (version 3.58.1) R (4.4.0) software package. Normalization factors were generated using the TMM method. Voom-normalized counts were analyzed using the lmFit and eBayes functions in Limma. The false discovery rate (FDR) was estimated using the Benjamini–Hochberg method. Gene set enrichment analysis (GSEA) was performed using curated signatures from MSigDB (v7.5). Ranking of genes was calculated using negative log10(*p*-value)*log2(fold change), and a *p*-value for each gene set was estimated by comparing the observed enrichment score to that obtained from a null distribution computed from 1000 permutations of genes within gene sets. FDR was estimated as previously described [64]. Enrichment plots were populated using GSEA (v3.0, Broad Institute, Cambridge, MA, USA) comparing control HL-60 and Molm-14 samples with HL-60 and Molm-14 samples treated with high concentrations of DSA. 

## 5. Conclusions

Frontline induction therapy is effective at achieving complete remission in some AML patients. However, several factors contribute to high mortality among AML patients, including resistance to the current standard-of-care chemotherapy regimen, resulting in high rates of relapse and low disease-free survival rates [65,66,67]. As a result, there is a continued need for the evaluation of chemotherapeutic agents that are efficacious in order to treat AML patients and improve their overall survival. In this study, we showed enhanced sensitivity of AML cells to DSA treatment. More specifically, DSA effectively decreased proliferation and induced apoptosis of AML cells within the picomolar range. Additionally, we observed the dysregulation of genes that mediate cellular processes such as G2/M checkpoint, DNA repair and apoptosis. In summary, our findings provide evidence of DSA’s efficacy on AML cells and support further investigation of this molecule as a potential therapeutic candidate for AML.

## Figures and Tables

**Table 1:** **Expression Patterns of Chemoresistance Genes in AML Cells.** The table provides a list of select genes expressed in HL-60 and Molm-14 cells after treatment with DSA that have been associated with chemoresistance in other cancers. The log2 fold change (Log2FC) for the expression of each gene in the HL-60 and Molm-14 cells are also provided.

Gene Symbol	Log2FC (HL-60)	Log2FC (Molm-14)
*GDF15*	6.84	1.17
*THBS1*	5.79	1.88
*CDKN1A*	5.47	1.65
*CLU*	4.71	1.19

## Data Availability

The datasets that were generated, analyzed and used in the current study are available from the corresponding author. RNA seq data that were generated are available online via the Gene Expression Omnibus (GEO) database managed by the National Center for Biotechnology Information (NCBI). The details can be accessed using the following link: https://www.ncbi.nlm.nih.gov/geo/query/acc.cgi?&acc=GSE245161&token=srqzkkakjdaltst (accessed on 23 February 2024). The details of this dataset are not to be shared or distributed without permission from the corresponding author. To facilitate this, the link is currently set to private until 10 October 2027, or further permission.

## Supplementary Materials

The supporting information can be downloaded at: https://www.mdpi.com/article/10.3390/ijms25084342/s1.
