# Supplementary material for: Duocarmycin SA Reduces Proliferation and Increases Apoptosis in Acute Myeloid Leukemia Cells In Vitro
Source: Int J Mol Sci. 2024 Apr 14;25(8):4342. doi: 10.3390/ijms25084342 (PMC11050052; doi:10.3390/ijms25084342)
Supplement: Supplementary file 1 [file ijms-25-04342-s001.zip › ijms-2909214-supplementary.pdf]

Supplemental Information

Duocarmycin SA Reduces Proliferation and Increases Apoptosis in Acute Myeloid Leukemia Cells In Vitro

William A. Chen [1], Terry G. Williams [1], Leena So [1], Natalie Drew [1], Jie Fang [2], Pedro Ochoa [3,4], Nhi Nguyen [1], Yasmeen Jawhar [1], Jide Otiji [1], Penelope J. Duerksen-Hughes [3], Mark E. Reeves [3,5], Carlos A. Casiano [3,4], Hongjian Jin [6], Sinisa Dovat [7], Jun Yang [2], Kristopher E. Boyle [1] and Olivia L. Francis-Boyle [1,3,8,*]

[1] Department of Pharmaceutical Sciences, School of Pharmacy, Loma Linda University, Shryock Hall 24745 Stewart Street, Loma Linda, CA 92350, USA
[2] Department of Surgery, St. Jude Children's Research Hospital, 262 Danny Thomas Place, Memphis, TN 38105, USA
[3] Department of Basic Sciences, School of Medicine, Loma Linda University, 11175 Campus Street, Loma Linda, CA 92350, USA
[4] Department of Surgery, School of Medicine, Loma Linda University, 11234 Anderson Street, Loma Linda, CA 92354, USA
[5] Center for Health Disparities and Molecular Medicine, Loma Linda University, 11085 Campus Street, Loma Linda, CA 92350, USA
[6] Center for Applied Bioinformatics, St. Jude Children's Research Hospital, 262 Danny Thomas Place, Memphis, TN 38105, USA
[7] Departments of Pediatrics, Biochemistry and Molecular Biology, and Pharmacology, Penn State Cancer Institute, 400 University Drive, Hershey, PA 17033, USA
[8] Department of Pathology and Human Anatomy, Division of Anatomy, School of Medicine, Loma Linda University, 11175 Campus Street, Loma Linda, CA 92350, USA
* Correspondence: ofrancis@llu.edu; Tel.: +1-909-558-9473 (ext. 49473)

This file contains supplementary figures and tables with legends.

Supplemental Table S1:

Antibody	Fluorochrome	Clone	Species Reactivity	Company
CD4	PE	A161A1	Human	Biolegend
CD8	PerCP	SK1	Human	Biolegend
CD13	PE	WM15	Human	Biolegend
CD14	PE/Cy7	HCD14	Human	Biolegend
CD19	APC	4G7	Human	Biolegend
CD19	PE/Cy7	HIB19	Human	Biolegend
CD33	APC	P67.6	Human	Biolegend
CD34	PerCP/Cy5.5	561	Human	Biolegend
CD45	FITC	H130	Human	Biolegend
CD45	PerCP	2D1	Human	Biolegend
CD117	FITC	104D2	Human	Biolegend

Supplemental Table S1: List of Antibodies used for Immunophenotyping AML cells. The list of antibodies, their clones and their fluorochromes are provided in the table above. Abbreviations: PE (Phycoerythrin); PerCP (Peridinin-Chlorophyll-Protein); PE/Cy7 (Phycoerythrin/Cyanine7); APC (Allophycocyanin); PerCP/Cy5.5 (Peridinin-Chlorophyll-Protein/Cyanine5.5); FITC (Fluorescein Isothiocyanate).

Supplemental Figure S1:

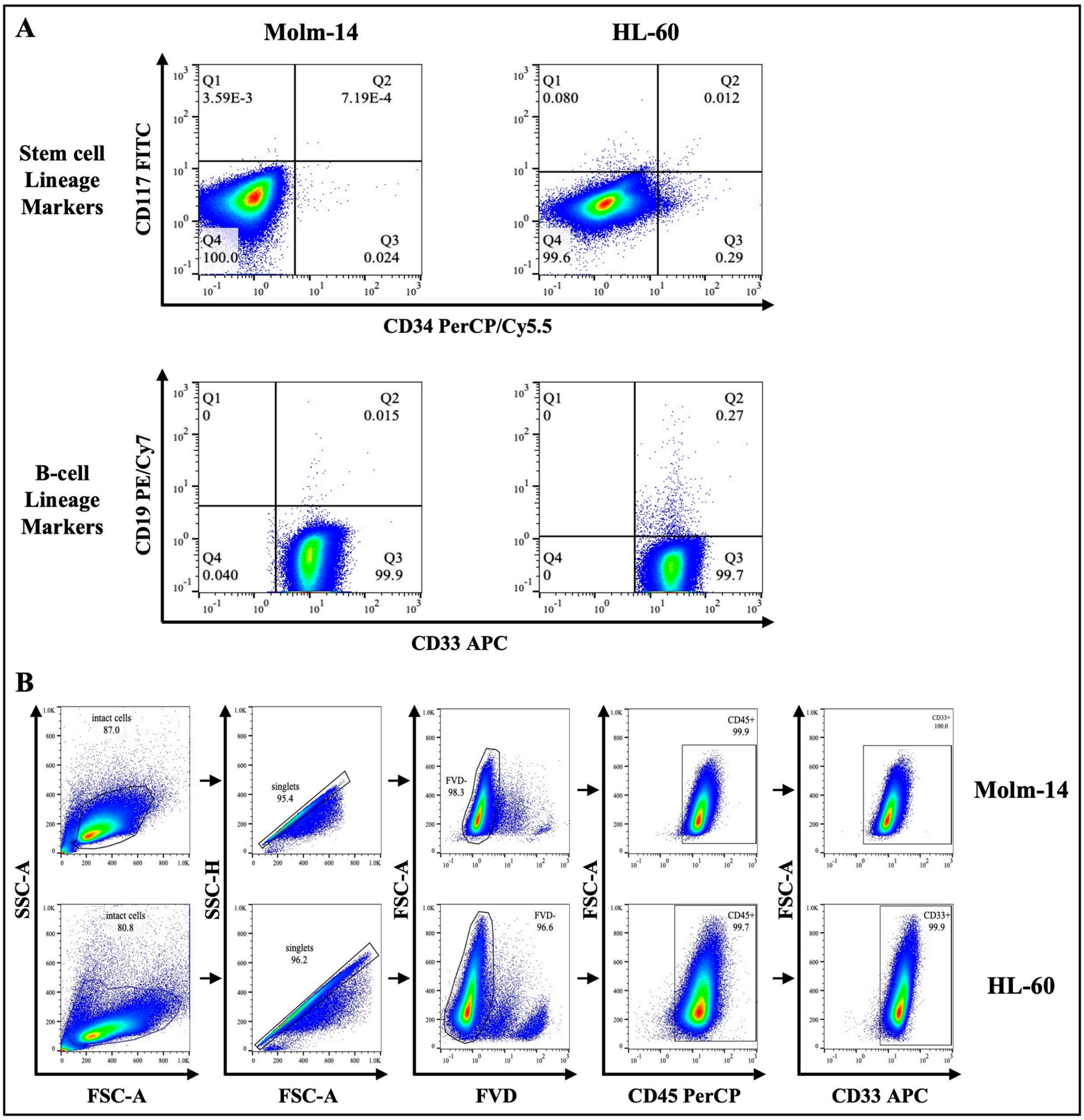

Supplemental Figure S1: Immunophenotyping of AML Cells and Gating Strategy. (A) Shown are representative dot plots of AML cells stained with the following markers: CD34, CD117 (stem cell lineage) and CD19 (B-cell lineage). **(B)** Prior to confirming the lineage markers, the gating strategy used to analyze the data included the creation of an intact cell gate (exclusion of debris), a singlet gate (exclusion of doublets using doublet discrimination), and a viable cell gate (exclusion of dead cells using a fixable viability dye). Leukocytes were identified by the expression of CD45 and myeloid cells by the expression of CD33.

Supplemental Figure S2A:

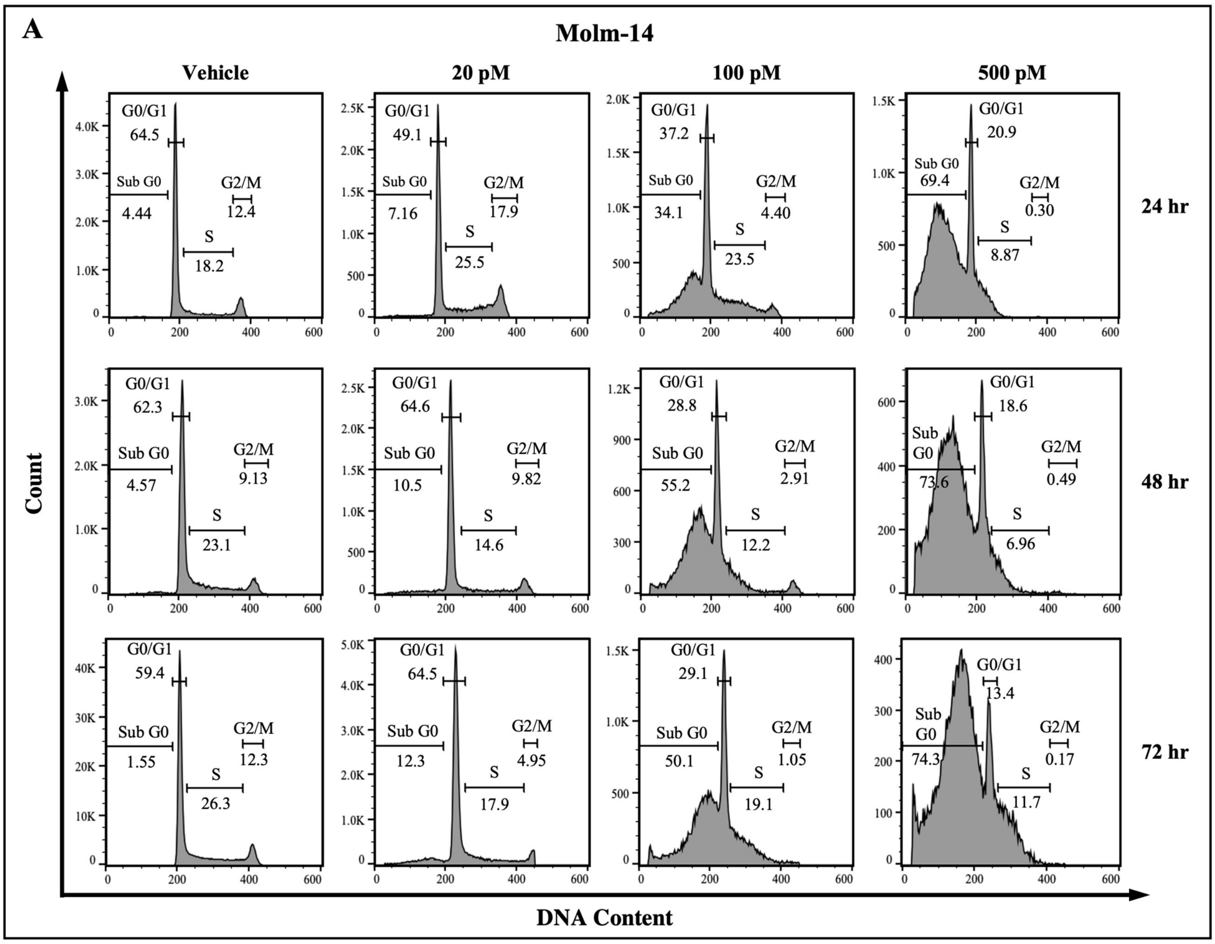

Supplemental Figure S2A: DSA Induces Cell Cycle Arrest in AML cells in Multiple Phases. Molm-14 cells were plated at 300,000 cells per well and incubated with vehicle (DMSO) or increasing concentrations of DSA (20, 100 and 500 pM). Cells were harvested at 24, 48 and 72 hours, stained with propidium iodide to identify cells in different phases of the cell cycle and analyzed by flow cytometry. G0/G1: Growth 0/Growth 1, S: Synthesis, G2/M: Growth 2/Mitosis, and sub G0: sub-Growth 0 (apoptotic cells). Histograms are representative of one of three independent experiments.

Supplemental Figure S2B:

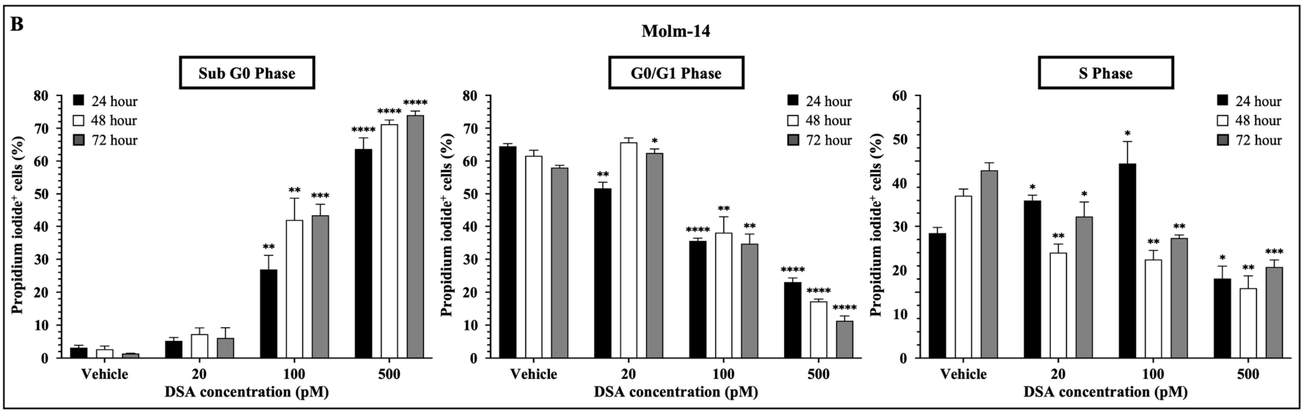

Supplemental Figure S2B: DSA Induces Cell Cycle Arrest in AML cells in Multiple Phases. Shown in Panel B is the quantification of the propidium iodide+ **Molm-14 cells** that are present in the Sub G0, G0/G1 and S phases of the cells' cycle. G0/G1: Growth 0/Growth 1, S: Synthesis, G2/M: Growth 2/Mitosis, and sub G0: sub-Growth 0 (apoptotic cells). The bar graphs were generated using GraphPad prism. Data shown is represented as the mean ± SEM that is representative of 3 independent experiments for each cell line. *p < 0.05, **p < 0.01, ***p <0.001, ****p < 0.0001

Supplemental Figure S2C:

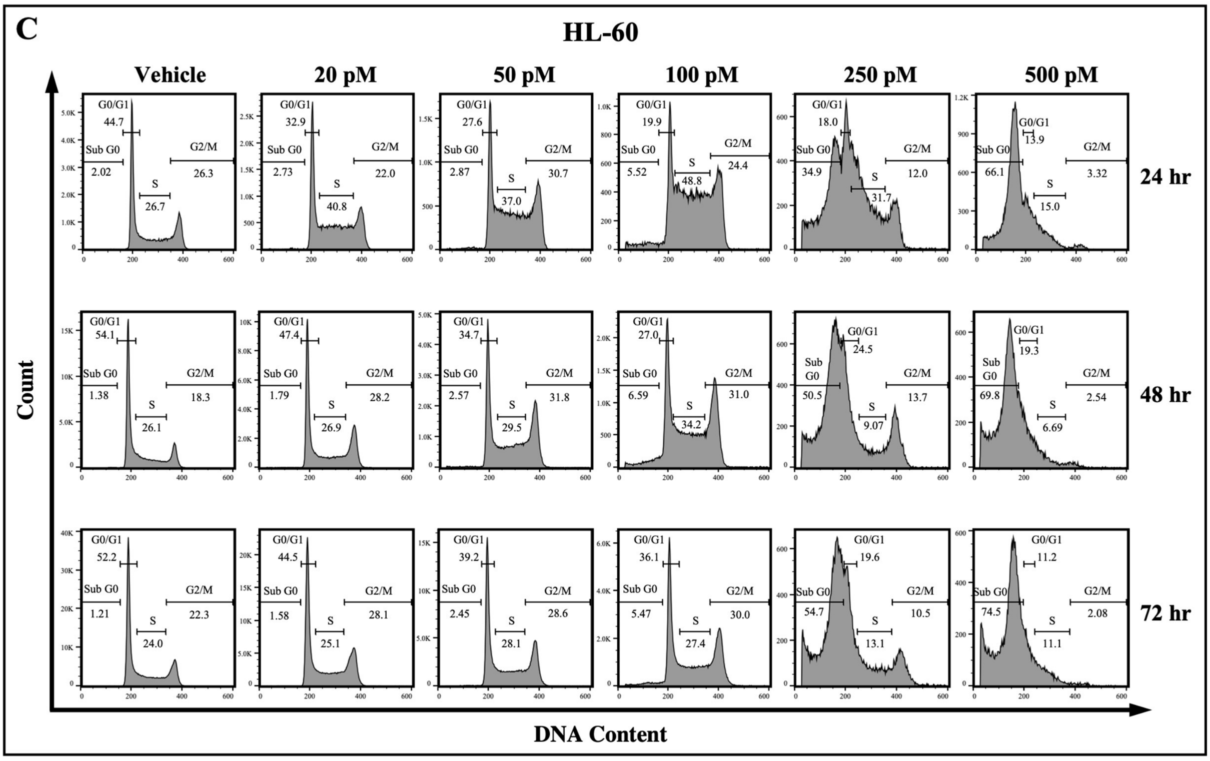

Supplemental Figure S2C: DSA Induces Cell Cycle Arrest in AML cells in Multiple Phases. HL-60 cells were plated at 150,000 cells per well and incubated with vehicle (DMSO) or increasing concentrations of DSA (20, 50, 100, 250 and 500 pM). Cells were harvested at 24, 48 and 72 hours, stained with propidium iodide to identify cells in different phases of the cell cycle and analyzed by flow cytometry. G0/G1: Growth 0/Growth 1, S: Synthesis, G2/M: Growth 2/Mitosis, and sub G0: sub-Growth 0 (apoptotic cells). Histograms are representative of one of three independent experiments.

Supplemental Figure S2D:

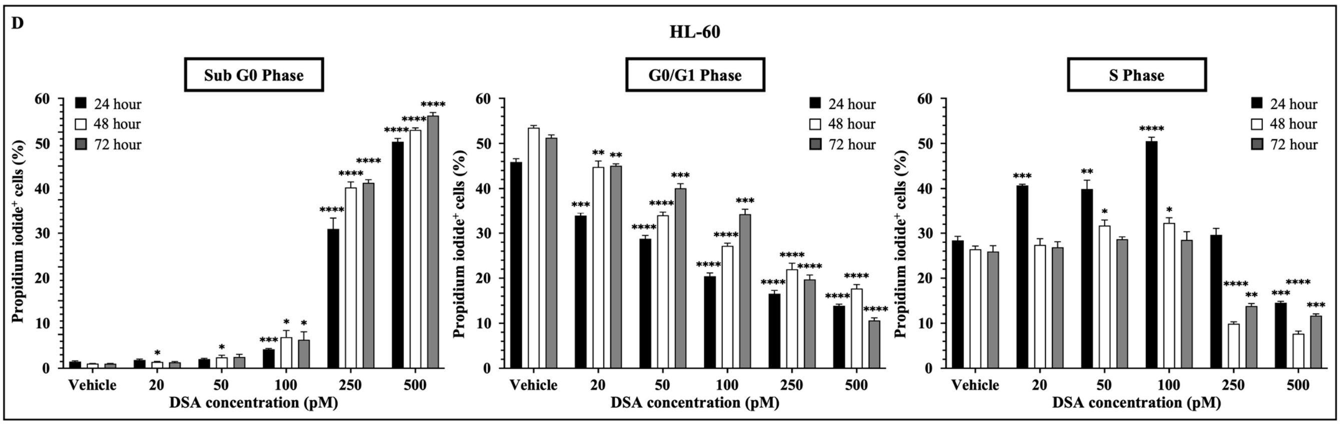

Supplemental Figure S2D: DSA Induces Cell Cycle Arrest in AML cells in Multiple Phases. Shown in Panel D is the quantification of the propidium iodide+ **Molm-14 cells** that are present in the Sub G0, G0/G1 and S phases of the cells' cycle. G0/G1: Growth 0/Growth 1, S: Synthesis, G2/M: Growth 2/Mitosis, and sub G0: sub-Growth 0 (apoptotic cells). The bar graphs were generated using GraphPad prism. Data shown is represented as the mean ± SEM that is representative of 3 independent experiments for each cell line. *p < 0.05, **p < 0.01, ***p <0.001, ****p < 0.0001

Supplemental Figure S3:

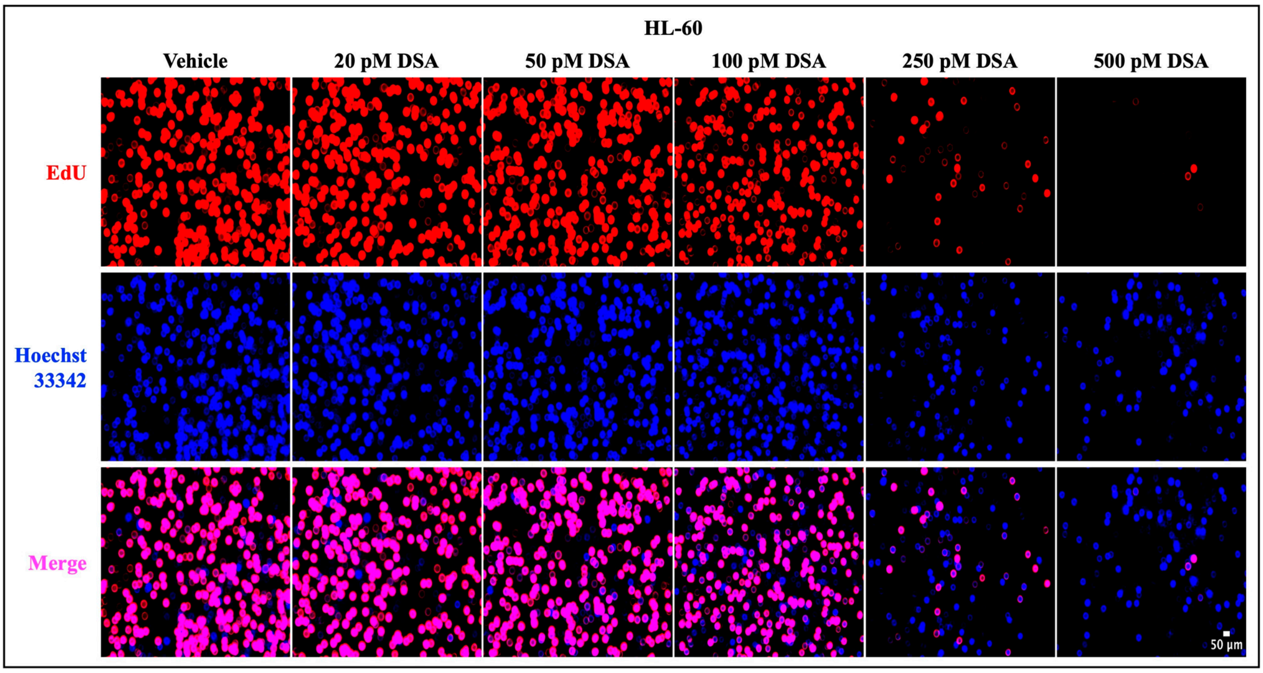

Supplemental Figure S3: DSA Decreases the Proliferation of AML Cells. HL-60 cells were plated at ~53,000 cells per well, incubated with vehicle (DMSO) or increasing concentrations of DSA (20, 50, 100, 250, and 500 pM) for 4 days, and treated with EdU for 3 days. Cells were harvested, fixed, and permeabilized prior to sequential staining with the Alexa Fluor 647 dye and Hoechst 33342. Wells were imaged using fluorescence microscopy at a total magnification of 100x. (A) Panel A shows images of HL-60 cells that are representative of one of three independent experiments.

Supplemental Figure S4A:

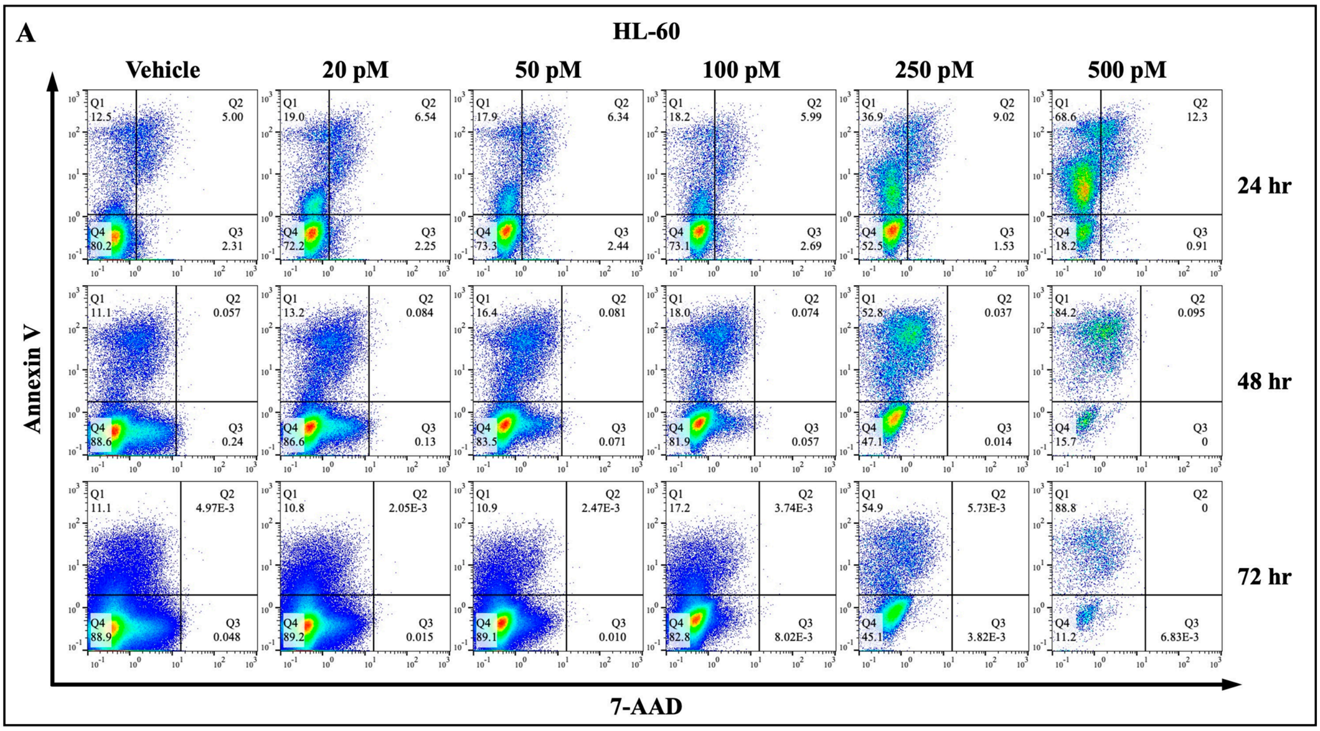

Supplemental Figure S4A: DSA Induces Early and Late-stage Apoptosis in AML Cells. HL-60 cells were plated at 150,000 cells per well and incubated with vehicle (DMSO) or 20, 100 and 500 pM of DSA. Cells were harvested at 24, 48 and 72 hours, stained with Annexin V and 7-AAD to detect apoptotic cells and analyzed by flow cytometry**.** (A) Panel A shows representative dot plots of early stage (Q1: Annexin V^{+}) and late stage (Q2: Annexin V^{+}7-AAD^{+}) apoptotic cells after treatment with DSA at the various time points.

Supplemental Figure S4B:

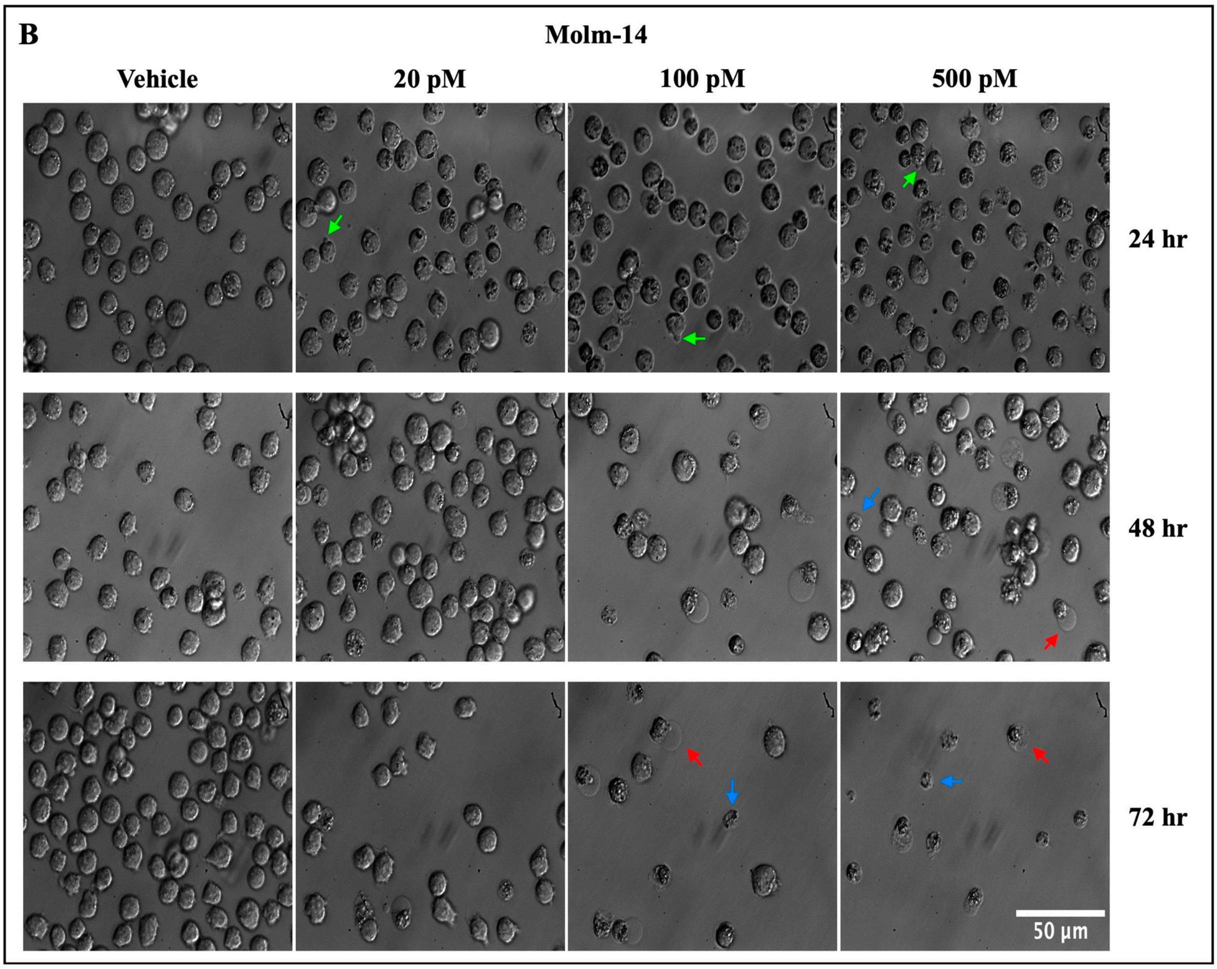

Supplemental Figure S4B: DSA Induces Morphological Changes as Evidence of Apoptosis in Molm-14 Cells. Molm-14 cells were plated at 300,000 cells per well and incubated with vehicle (DMSO) or increasing concentrations of DSA (20, 100 and 500 pM). Cells were imaged at 24, 48, and 72-hours using Hoffman Modulation phase contrast microscopy at a total magnification of 400x. Apoptotic blebs (green arrows), ghost membranes (red arrows) and cell shrinkage (blue arrows) were observed. Representative images shown are from one of three independent experiments.

Supplemental Figure S4C:

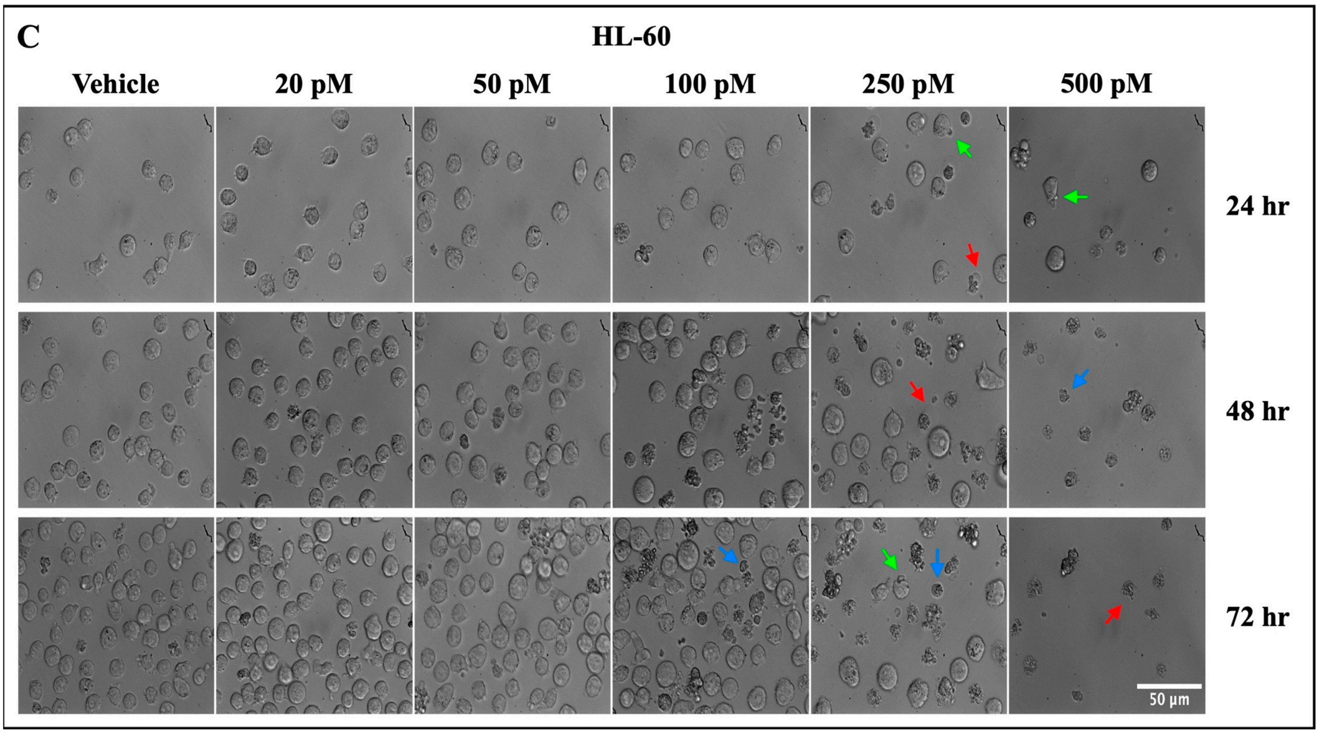

Supplemental Figure S4C: DSA Induces Morphological Changes as Evidence of Apoptosis in HL-60 Cells. HL-60 cells were plated at 150,000 cells per well and incubated with vehicle (DMSO) or increasing concentrations of DSA (20, 50, 100, 250 and 500 pM). Cells were imaged at 24-, 48-, and 72-hours using Hoffman Modulation phase contrast microscopy at a total magnification of 400x. Apoptotic blebs (green arrows), ghost membranes (red arrows) and cell shrinkage (blue arrows) were observed. Representative images shown are from one of two independent experiments.

Supplemental Table S2A:

Molm-14 Upregulated Genes			
**Gene ID**	**Gene Symbol**	**Gene description**	**log2FC**
ENSG00000176020.8	*AMIGO3*	Adhesion molecule with Ig like domain 3	6.79
ENSG00000267303.1	*AC011511.4*	Unknown (novel transcript)	5.34
ENSG00000273513.1	*TBC1D3K*	TBC1 domain family member 3K	5.11
ENSG00000272196.2	*HIST2H2AA4*	Histone cluster 2 H2A family member a4	5.08
ENSG00000130635.15	*COL5A1*	Collagen type V alpha 1 chain	4.66
ENSG00000267697.2	*LUZP6*	Leucine zipper protein 6	4.24
ENSG00000133063.16	*CHIT1*	Chitinase 1	4.21
ENSG00000283900.1	*TPTEP2-CSNK1E*	TPTEP2-CSNK1E readthrough	4.16
ENSG00000169885.10	*CALML6*	Calmodulin like 6	3.98
ENSG00000235098.8	*ANKRD65*	Ankyrin repeat domain 65	3.98
ENSG00000268193.5	*AC002985.1*	Unknown (novel transcript)	3.69
ENSG00000205236.6	*AC105052.1*	Unknown (novel protein)	3.66
ENSG00000121068.14	*TBX2*	T-box transcription factor 2	3.60
ENSG00000168427.9	*KLHL30*	Kelch like family member 30	3.42
ENSG00000257524.6	*AL157935.2*	Unknown (novel protein)	3.38
ENSG00000038427.16	*VCAN*	Versican	3.36
ENSG00000182687.4	*GALR2*	Galanin receptor 2	3.33
ENSG00000161544.10	*CYGB*	Cytoglobin	3.26
ENSG00000155428.12	*TRIM74*	Tripartite motif containing 74	3.25
ENSG00000138131.4	*LOXL4*	Lysyl oxidase like 4	3.20

Supplemental Table S2A: Top 20 Upregulated Genes in Molm-14 Cells. The list of the top 20 genes that are upregulated in Molm-14 cells after treatment with DSA for 36 hours.

Supplemental Table S2B:

Molm-14 Downregulated Genes			
**Gene ID**	**Gene Symbol**	**Gene description**	**log2FC**
ENSG00000285000.1	*AC008581.2*	Unknown (novel protein)	-6.12
ENSG00000258555.6	*SPECC1L-ADORA2A*	SPECC1L-ADORA2A readthrough (NMD candidate)	-5.75
ENSG00000285283.1	*AL035078.4*	Unknown (novel protein)	-5.38
ENSG00000285238.2	*AC006064.6*	Unknown (novel transcript)	-5.15
ENSG00000124194.16	*GDAP1L1*	Ganglioside induced differentiation associated protein 1 like 1	-4.73
ENSG00000203812.2	*HIST2H2AA3*	Histone cluster 2 h2a family member a3	-4.53
ENSG00000196517.11	*SLC6A9*	Solute carrier family 6 member 9	-4.23
ENSG00000107159.13	*CA9*	Carbonic anhydrase 9	-4.14
ENSG00000186150.4	*UBL4B*	Ubiquitin like 4B	-3.94
ENSG00000184005.11	*ST6GALNAC3*	ST6 N-acetylgalactosaminide alpha-2,6-sialyltransferase 3	-3.93
ENSG00000131152.4	*AC010531.1*	Unknown (novel protein)	-3.79
ENSG00000204805.9	*AL391987.2*	Unknown	-3.78
ENSG00000260537.2	*AC012184.2*	Unknown (novel protein)	-3.59
ENSG00000116191.17	*RALGPS2*	Ral GEF with PH domain and SH3 binding motif 2	-3.39
ENSG00000179813.7	*FAM216B*	Family with sequence similarity 216 member B	-3.27
ENSG00000273217.1	*AC008695.1*	Unknown (novel protein)	-3.22
ENSG00000131711.15	*MAP1B*	Microtubule associated protein 1B	-3.00
ENSG00000183682.8	*BMP8A*	Bone morphogenetic protein 8a	-2.80
ENSG00000139269.3	*INHBE*	Inhibin subunit beta E	-2.76
ENSG00000128965.13	*CHAC1*	ChaC glutathione specific gamma-glutamylcyclotransferase 1	-2.73

Supplemental Table S2B: Top 20 Downregulated Genes in Molm-14 Cells. The list of the top 20 genes that are downregulated in Molm-14 cells after treatment with DSA for 36 hours.

Supplemental Table S2C:

HL-60 Upregulated Genes			
**Gene ID**	**Gene Symbol**	**Gene description**	**log2FC**
ENSG00000285245.1	*AL162417.1*	Unknown (novel protein)	8.11
ENSG00000264230.9	*ANXA8L1*	Annexin A8 like 1	6.93
ENSG00000130513.6	*GDF15*	Growth differentiation factor 15	6.84
ENSG00000079393.20	*DUSP13*	Dual specificity phosphatase 13	6.55
ENSG00000135480.16	*KRT7*	Keratin, type II cytoskeletal 7	6.35
ENSG00000204099.11	*NEU4*	Neuraminidase 4	6.27
ENSG00000105419.17	*MEIS3*	Meis homeobox 3	6.26
ENSG00000130487.8	*KLHDC7B*	Kelch domain containing 7B	6.24
ENSG00000176046.8	*NUPR1*	Nuclear protein 1	6.22
ENSG00000099957.16	*P2RX6*	Purinergic receptor P2X 6	6.21
ENSG00000113302.4	*IL12B*	Interleukin 12B	6.12
ENSG00000198125.13	*MB*	Myoglobin	6.10
ENSG00000186510.12	*CLCNKA*	Chloride voltage-gated channel Ka	6.04
ENSG00000118257.16	*NRP2*	Neuropilin 2	5.79
ENSG00000137801.10	*THBS1*	Thrombospondin 1	5.79
ENSG00000041982.16	*TNC*	Tenascin C	5.79
ENSG00000070808.15	*CAMK2A*	Calcium/calmodulin dependent protein kinase II alpha	5.74
ENSG00000092051.17	*JPH4*	Junctophilin 4	5.57
ENSG00000115414.19	*FN1*	Fibronectin 1	5.55
ENSG00000205277.9	*MUC12*	Mucin 12	5.53

Supplemental Table S2C: Top 20 Upregulated Genes in HL-60 Cells. The list of the top 20 genes that are upregulated in HL-60 cells after treatment with DSA for 36 hours.

Supplemental Table S2D:

HL-60 Downregulated Genes			
**Gene ID**	**Gene Symbol**	**Gene description**	**log2FC**
ENSG00000276612.3	*FP565260.2*	Unknown (novel protein)	-6.26
ENSG00000284057.1	*AP001273.2*	Unknown (novel protein)	-5.91
ENSG00000267697.2	*LUZP6*	Leucine zipper protein 6	-5.66
ENSG00000256349.1	*AP002748.4*	Unknown (novel protein)	-4.95
ENSG00000286221.1	*AC009070.1*	Unknown (novel protein)	-4.70
ENSG00000123892.12	*RAB38*	Ras-related protein 38	-4.64
ENSG00000196826.7	*AC008758.1*	Unknown (novel zinc finger protein)	-4.36
ENSG00000258984.5	*UBE2F-SCLY*	UBE2F-SCLY readthrough (NMD candidate)	-4.32
ENSG00000257921.6	*AC025165.3*	Unknown (novel protein)	-4.27
ENSG00000283930.1	*AL117339.5*	PLD5 pseudogene 1	-4.23
ENSG00000206047.2	*DEFA1*	Defensin alpha 1	-3.90
ENSG00000285547.1	*AL133500.1*	Unknown (novel protein)	-3.84
ENSG00000256861.1	*AC048338.1*	Unknown (novel protein)	-3.69
ENSG00000257411.2	*AC034102.3*	Unknown (novel protein)	-3.68
ENSG00000283149.1	*AC068631.2*	Unknown (novel protein)	-3.60
ENSG00000272968.5	*RBAK-RBAKDN*	RBAK-RBAKDN readthrough	-3.51
ENSG00000091137.13	*SLC26A4*	Solute carrier family 26 member 4	-3.51
ENSG00000273294.1	*C1QTNF3-AMACR*	C1QTNF3-AMACR readthrough (NMD candidate)	-3.40
ENSG00000273003.1	*ARL2-SNX15*	ARL2-SNX15 readthrough (NMD candidate)	-3.38
ENSG00000261582.1	*AL121753.1*	Unknown (novel transcript)	-3.38

Supplemental Table S2D: Top 20 Downregulated Genes in HL-60 Cells. The list of the top 20 genes that are downregulated in HL-60 cells after treatment with DSA for 36 hours.

Supplemental Figure S5A-C:

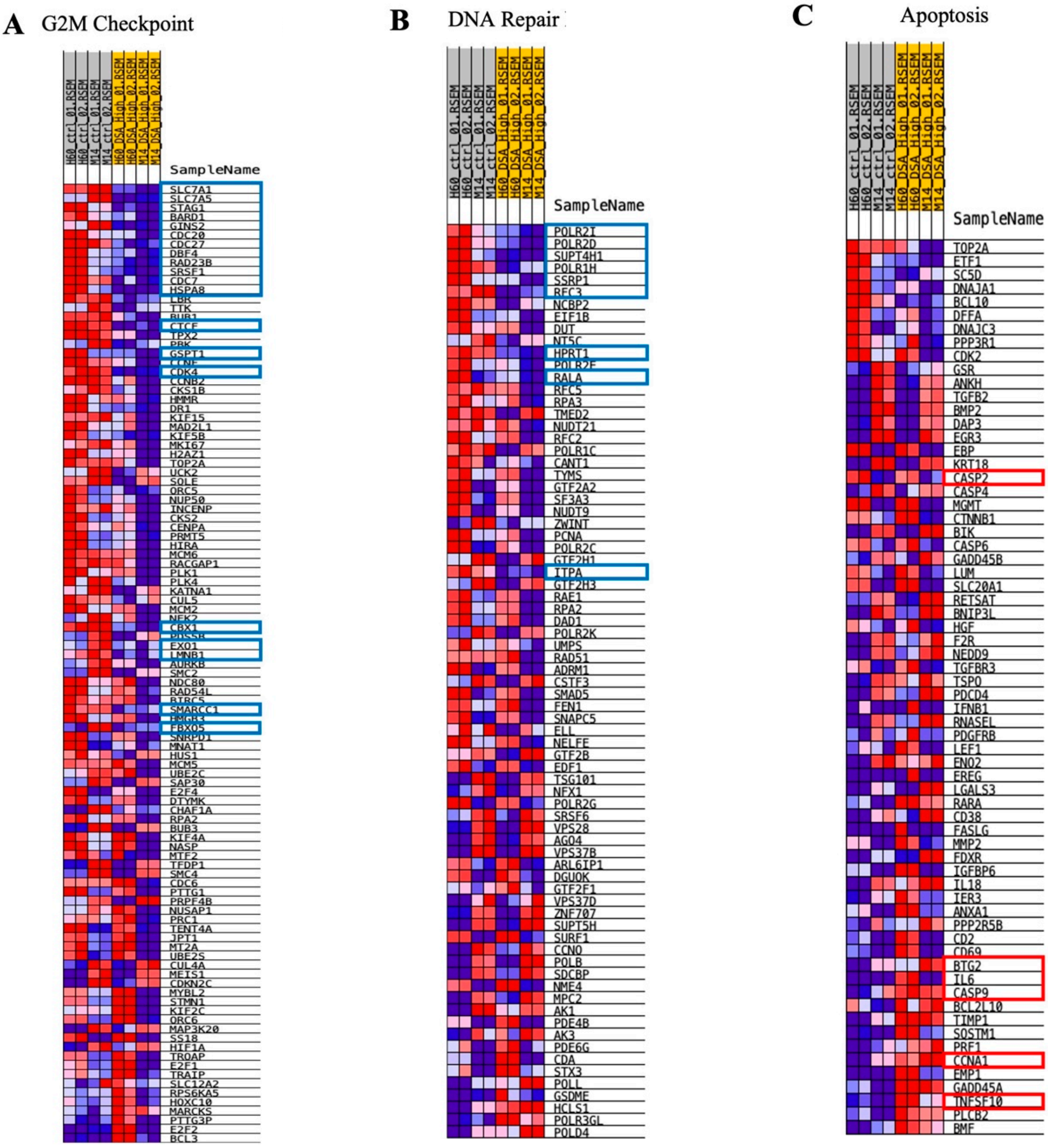

Supplemental Figure S5A-C: Heatmap Showing Individual Genes found in Enriched Gene Sets from GSEA. The figure shows the list of genes that were identified in three Hallmark Gene Sets: G2/M Checkpoint (A), DNA Repair (B) and Apoptosis (C). Highlighted with boxes are the genes that were downregulated (blue boxes) or upregulated (red boxes) in each gene set.

Supplemental Table S3:

HL-60 Untreated vs Molm-14 Untreated						HL-60 Treated vs Molm-14 Treated					
**Gene Symbol**	**DNA Repair Pathway**	**HL-60 Expression**		**Molm-14 Expression**		**Gene Symbol**	**DNA Repair Pathway**	**HL-60 Expression**		**Molm-14 Expression**	
POLR2E	NER					RFC5	MMR, NER				
POLR2C	NER					RFC2	MMR, NER				
POLR2K	NER					PCNA	BER, MMR, NER				
POLB	BER					POLR2C	NER				
						GTF2H1	NER				
						GTF2H3	NER				
						RPA2	MMR, NER				
						POLR2K	NER				
						RAD51	HR				
						FEN1	BER				
						POLB	BER				
						POLL	BER, HR, NHEJ				

Supplemental Table S3: Expression of DNA Repair Genes and Associated DNA Repair Pathways in AML Cells before and after Treatment with DSA. The table provides a list of genes, their associated DNA repair pathways and their expression profiles in Molm-14 and HL-60 cells before and after treatment with DSA. The differential expression of DNA repair genes in the untreated groups prior to treatment with DSA are provided in the table (Left) and the differential expression of DNA repair genes in the treated groups after treatment with DSA are provided in the table (Right). DNA repair pathways key: base excision repair (BER), nucleotide excision repair (NER), mismatch repair (MMR), homologous recombination (HR) and non-homologous end joining (NHEJ). Gene expression pattern: blue- downregulated; red- upregulated.
